# Preserved Left Ventricular Function despite Myocardial Fibrosis and Myopathy in the Dystrophin-Deficient D2.B10-Dmd*^mdx^*/J Mouse

**DOI:** 10.1155/2022/5362115

**Published:** 2022-03-16

**Authors:** Holly M. Hayes, Julie Angerosa, Adam T. Piers, Jason D. White, Jane Koleff, Madeline Thurgood, Jessica Moody, Michael M. Cheung, Salvatore Pepe

**Affiliations:** ^1^Heart Research, Murdoch Children's Research Institute, Royal Children's Hospital, 50 Flemington Road, Parkville, Melbourne, VIC 3052, Australia; ^2^Department of Paediatrics, University of Melbourne, Melbourne, Australia; ^3^Faculty of Veterinary and Agricultural Science, University of Melbourne, Melbourne, Australia

## Abstract

Duchenne muscular dystrophy involves an absence of dystrophin, a cytoskeletal protein which supports cell structural integrity and scaffolding for signalling molecules in myocytes. Affected individuals experience progressive muscle degeneration that leads to irreversible loss of ambulation and respiratory diaphragm function. Although clinical management has greatly advanced, heart failure due to myocardial cell loss and fibrosis remains the major cause of death. We examined cardiac morphology and function in D2.B10-Dmd*^mdx^*/J (D2-*mdx*) mice, a relatively new mouse model of muscular dystrophy, which we compared to their wild-type background DBA/2J mice (DBA/2). We also tested whether drug treatment with a specific blocker of mitochondrial permeability transition pore opening (Debio-025), or ACE inhibition (Perindopril), had any effect on dystrophy-related cardiomyopathy. D2-*mdx* mice were treated for six weeks with Vehicle control, Debio-025 (20 mg/kg/day), Perindopril (2 mg/kg/day), or a combination (*n* = 8/group). At 18 weeks, compared to DBA/2, D2-*mdx* hearts displayed greater ventricular collagen, lower cell density, greater cell diameter, and greater protein expression levels of IL-6, TLR4, BAX/Bcl2, caspase-3, PGC-1*α*, and notably monoamine oxidases A and B. Remarkably, these adaptations in D2-*mdx* mice were associated with preserved resting left ventricular function similar to DBA/2 mice. Compared to vehicle, although Perindopril partly attenuated the increase in heart weight and collagen at 18 weeks, the drug treatments had no marked impact on dystrophic cardiomyopathy.

## 1. Introduction

Duchenne muscular dystrophy (DMD) is an X chromosome-linked muscle-wasting disease that affects approximately 1 in 3600-6000 boys [1, 2]. A mutation in the dystrophin gene prevents synthesis of functional dystrophin protein resulting in mechanical and molecular signalling-related cell injury and mitochondria-related cell death in skeletal and cardiac muscle [3–8]. Gene and cell-based therapies for directly restoring dystrophin are clinically unavailable due to current technological limitations. Notably, clinical management with corticosteroids, manually assisted coughing devices, and mechanical ventilation has greatly extended patient survival to adulthood [9, 10]. Cardiomyopathic heart failure now prevails for these patients, with most presenting cardiac-related complications by the age of 20 [11–13]. The current standard of care recommended by the DMD Care Considerations Working Group to delay the onset of cardiomyopathy includes the use of angiotensin-converting enzyme (ACE) inhibitors (i.e., Perindopril) by 10 years of age [2]. Early ACE inhibitor therapy has been shown to preserve cardiac function in patients with DMD and “*mdx*” mouse models [14, 15].

Myocardial fibrosis is one of the earliest clinically detectable cardiac features of DMD that contributes to the maladaptive remodelling [16]. Cardiomyocytes demonstrate membrane instability and dysregulated Ca^2+^ handling which augments Ca^2+^ influx, leading to the formation of fibrosis and cellular necrosis [17, 18]. Aside from increasing the stiffness of the myocardium and impeding its ability to contract and relax, myocardial fibrosis promotes compensatory hypertrophy of neighbouring cardiomyocytes that disrupts the electrical conduction and coupling system in the heart, thus increasing the risk of serious arrhythmias [19–21]. As the onset of fibrosis begins early in DMD, slowing the progression of myocardial fibrosis is an important therapeutic target to delay the onset of cardiomyopathy.

Mitochondrial dysfunction is another potential therapeutic target in DMD. Diminished mitochondrial energy production, Ca^2+^ dysregulation, oxidative stress, and mitochondrial-mediated cell death underlie muscle degeneration associated with DMD cardiomyopathy [6, 7, 17, 22–24]. Impairment of mitochondrial respiration and prolonged increases in cytosolic Ca^2+^ contribute to opening of the mitochondrial permeability transition pore (mPTP) and subsequent mitochondrial-dependent cell death signalling [25]. The mPTP is a large, voltage-dependent channel in the inner mitochondrial membrane that opens with inner membrane depolarization [ 26, 27]. Prolonged, irreversible openings of the mPTP have been linked to ischemic cell death associated with stroke, myocardial infarction, and cardiomyopathy associated with DMD [28]. An overload of Ca^2+^ in the mitochondrial matrix and excessive production of ROS contribute to the persistent opening of the mPTP, inner mitochondrial membrane depolarization and loss of oxidative phosphorylation-dependent ATP production [26, 29–32], mitochondrial swelling, outer mitochondrial membrane rupture, oxidation of cardiolipin, release of cytochrome c, and proapoptotic protein activity [30, 33, 34]. Loss of mitochondria beyond a critical threshold ultimately causes cell death and exacerbation of myocardial complications in DMD [35]. Thus, a drug that prevents irreversible opening of mPTP may delay myocyte death in DMD. Debio-025 is a prolyl isomerase inhibitor that inhibits the mPTP-sensitizing protein, cyclophilin D, to prevent mitochondrial swelling and apoptosis [36]. Preliminary studies conducted with mouse models of DMD have shown that Debio-025 protects skeletal and diaphragm muscles from cell death, improves myofibril organisation, reduces mitochondrial swelling and fibrosis in skeletal muscle, and improves overall muscle function [24, 36, 37]. It has also been shown to improve skeletal muscle fibre attachment to the myotendinous junction, normalise the organisation and ultrastructure of cristae, and prevent mPTP opening in isolated mitochondria from DMD patients [38]. However, the effect of Debio-025 on heart function has not been examined in patients with DMD.

Mice featuring a mutation in the dystrophin gene (i.e., “*mdx*”) have been crossed with different genetic backgrounds to create a disease model that mimics the human condition. Complicating model development, the genetic background of wild-type mice has been shown to significantly affect the phenotype of a given single gene mutation due to the presence of modifier genes [11, 39]. Mounting evidence has shown that, compared to the most commonly used C57BL/10-*Dmd^mdx^* (BL10-*mdx*) on a C57BL/10ScSn background, the recently described D2.B10-*Dmd^mdx^*/J (D2-*mdx*) on a DBA2/J background displays a more pronounced dystrophic phenotype that is characterized by muscular atrophy, increased inflammation and fibrosis, and reduced skeletal muscle function as early as seven weeks of age [3, 40–43]. Using the *γ*-sarcoglycan- (*Sgcg*-) null mouse model of muscular dystrophy and cardiomyopathy on a DBA2/J background, a polymorphism in the coding region of the latent TGF-*β*-binding protein 4 gene (*Ltbp4*) resulting in a 12-amino-acid deletion in LTBP4 protein was found to be responsible for increased proteolysis, fibrosis, and atrophy in these mice [44]. As LTBP4 usually sequesters TGF-*β* to limiting signalling, importantly, polymorphism of the human *Ltbp4* locus has been shown to be a genetic modifier of disease severity in DMD patients [45, 46]. D2-*mdx* mice have also been shown to have a dysfunctional annexin A6 (*anxa6*) gene which is responsible for decreased self-renewal capacity of satellite cells [47]. D2-*mdx* mice also carry the dystrophic calcinosis 1 (*dyscalc1*) gene locus which contributes susceptibility to calcinosis in the heart and skeletal muscles [48]. Recently, study of the D2-*mdx* muscle phenotype has confirmed early presence of impaired mitochondrial function in the heart [25]; however, whether this has early impact on *in vivo* cardiac function that can be targeted by therapeutic intervention requires investigation. Thus, the aims of the current study were to characterize the extent of cardiac pathology and dysfunction in D2-*mdx* mice, compared to wild-type DBA/2J background strain, and to test the effects of ACE inhibition with Perindopril and/or mPTP blockade with Debio-025.

## 2. Materials and Methods

### 2.1. Animal Handling and Care

The study was approved by the Murdoch Children's Research Institute (MCRI) Animal Ethics Committee (A859) and was performed in accordance with the Australian Code of Practice for the Care and Use of Animals for Scientific Purposes (8th Edition, 2013). DBA/2J (referred to as DBA/2) male mice (*n* = 4) were purchased from the Animal Resources Centre (Western Australia, Australia), and D2.B10-Dmd*^mdx^*/J (D2-*mdx*) male mice (JAX stock #013141; *n* = 32) were purchased from Jackson Laboratory (Bar Harbor, ME). D2-*mdx* mice were bred from C57BL/10-*mdx* mice that carry a spontaneous mutation resulting in a stop codon at position 3185 within exon 23 of the dystrophin gene on the X chromosome and were backcrossed with DBA/2J inbred mice [3, 40, 49]. Mice were housed at a density of up to five males in individually ventilated cages (Techniplast Blue Line) maintained at 65-75 air changes per hour. Rooms were maintained at 18-24°C with dawn/dusk timed lighting. Mice had access to irradiated rodent diet (18% protein, Teklad) and water *ad libitum*. Animals were monitored and weighed daily.

### 2.2. Drug Treatment

D2-*mdx* mice were randomised into four treatment groups (*n* = 8/group) as follows: (1) Vehicle control (phosphate-buffered saline (PBS)+Debio-025 carrier), (2) Perindopril (2 mg/kg/day; P0094, Sigma-Aldrich, St. Louis, MO, USA), (3) Debio-025 (20 mg/kg/day; Alisporivir; Debiopharm Research & Manufacturing SA, Martigny, Switzerland), and (4) Perindopril (2 mg/kg/day)+Debio-025 (20 mg/kg/day). Treatments were delivered via oral gavage as a volume of 0.1 mL. Treatment began at 12 weeks of age for six weeks.

### 2.3. Echocardiography

Ultrasound imaging at 10 and 18 weeks (before and after treatment) was performed with a Vevo 3100 Ultrasound Machine (FUJIFILM VisualSonics, Canada) to obtain LV functional and structural measurements. Mice were anaesthetized with 4% isoflurane and maintained with 2% isoflurane/oxygen flow. Hair was removed from the chest with depilatory hair removal cream to facilitate ultrasound transmission gel (Aquasonic100, Parker Laboratories Inc., Fairfield, NJ) and ultrasound probe placement. Heart rate and rectal body temperature were monitored throughout the procedure. Two-dimensional brightness mode (B-mode) and motion mode (M-mode) images were acquired from a parasternal long axis and parasternal short axis LV view and used to obtain LV end-diastolic (LVED) dimension, LV end-systolic (LVES) dimension, interventricular septum (IVS) diameter during systole and diastole, and posterior wall thickness (PWT) during systole and diastole. LV mass, LV fractional shortening (FS), and LV ejection fraction (EF) (measures of systolic function) were calculated based on LV chamber diameter and volume measurements using VevoLAB Version 3.1.0 software package (FS = ((LVED diameter − LVES diameter)/LVED diameter) × 100; EF = ((LVED volume − LVES volume)/LVED volume) × 100).

### 2.4. Forelimb Grip Strength Test

As the skeletal muscle phenotype of this mouse model has been previously characterized and the current study focus was the heart, a single skeletal muscle function test was performed. Forelimb grip strength was measured *in vivo* at 10 and 18 weeks (before and after treatment) for DBA/2 and D2-mdx mice, using a grip strength meter (Columbus Instruments, Columbus, OH, USA) according to TREAT-NMD SOP DMD_M.2.2.001 protocol. Using a bar attached to an isometric force transducer, forelimb grip strength was measured as the amount of horizontal force produced to break the grip of a mouse, while suspended by its tail [50]. With a minute of rest between each pull, five consecutive measurements were recorded for each mouse and then averaged and normalised to body weight.

### 2.5. Tissue Collection

At 18 weeks of age, mice were anaesthetized with 4% isoflurane/oxygen flow. Once unconscious and assessments were complete, a cardiac puncture was performed to collect blood directly from the heart. Mice were then killed by cervical dislocation and dissected to obtain the heart, diaphragm, and tibia. All tissues were individually weighed, snap frozen in liquid N_2_, and stored at -80°C until required. Diaphragms and transverse heart slices were also fixed in 10% buffered formalin, then embedded in paraffin wax, sectioned at 3 *μ*m, and stained using Masson's trichrome to detect collagen as a measure of fibrosis [51].

Whole heart transverse sections were digitally imaged using a Mirax Digital Slide Scanner (3DHISTECH Ltd., Budapest, Hungary). Whole diaphragms were imaged using a Leica DM 1000 Light Microscope (Leica Microsystems, Wetzlar, Germany). Quantification of fibrosis was performed using ImagePro (Version 9.2). The percentage of fibrotic tissue was determined by dividing the total of the blue collagen areas by the total area. For each mouse, the RV, LV, and septum were also imaged (Leica DM1000 Light Microscope, Wetzlar, Germany) to determine cell density (cell count/area) and cell diameter. For each heart region, a total of 5 areas of size 150 × 130 *μ*m (that excluded regions of dystrophic calcinosis) were assessed for a minimum of 100 cells using ImageJ (https://imagej.nih.gov/ij/). Cell diameter measurements were performed using Feret's diameter in ImageJ.

### 2.6. Electron Microscopy

Electron microscopy was conducted on LV heart sections of D2-mdx mice from each treatment group to visualise differences in cellular and intracellular structure and organisation, particularly focusing on mitochondria. Excised myocardial samples were cut into ~1 mm cubed blocks and fixed in PBS with 2.5% cacodylate-buffered glutaraldehyde, 2% paraformaldehyde, and 1% osmium tetroxide for 24 hours. Samples were then rinsed in fresh PBS, dehydrated in an alcohol series, and embedded in Epon 812. Sections were examined with a Zeiss Libra 120 PLUS Energy-Filtered Transmission Electron Microscope (Zeiss, Oberkochen, Germany).

### 2.7. Protein Analysis

Western immunoblotting was performed using heart tissue frozen at necropsy to determine expression of key proteins as per Table 1. Protein was extracted from 50 mg of frozen heart tissue powder per mouse following addition of modified radioimmunoprecipitation assay (RIPA) buffer (0.5 mL) containing 50 mM Tris-HCl at pH 7.4 (15567-027, Thermo Fisher), 1% Nonyl Phenoxylpolethoxylethanol (Tergitol; NP40S, Sigma-Aldrich), 150 mM sodium chloride (NaCl; 1.06404.5000, Merck-Millipore, Burlington, MA), and 500 mM ethylenediaminetetraacetic acid (EDTA; T9285, Sigma-Aldrich). One tablet of protease inhibitor cocktail (05892970001, Roche, Basel, Switzerland) and one tablet of phosphatase cocktail (04906837001, Roche) were added per 10 mL of RIPA buffer. The mixture was vortexed for 60 seconds and placed on ice for 45 minutes. Samples were further homogenised via TissueRuptor (Qiagen, Hilden, Germany) in two bursts of 30 seconds' duration. Samples were centrifuged at 2000 × *g* for 10 minutes at 4°C, and supernatants were collected and transferred into a fresh tube. A bicinchoninic acid (BCA) assay kit (B9643, Sigma-Aldrich) was used to determine total protein concentration as per manufacturer's protocol (A2153, Sigma-Aldrich). All samples were diluted 1 : 25 with nuclease free water (9937, Ambion, Austin, TX), and 10 *μ*L aliquots were pipetted in triplicate into a 96-well spectrophotometric plate, with 80 *μ*L of BCA. The plate was then incubated (23°C, 30 minutes), and then, optical density was determined at 560 nm with a Fluostar Optima spectrophotometric plate reader (BMG LabTech, Victoria, Australia).

Bax = Bcl-2-associated X; Bcl-2 = B cell lymphoma 2; GAPDH = glyceraldehyde 3-phosphate dehydrogenase; LC3B = light chain 3B; MAO = monoamine oxidase A and B; NF-*κ*B = nuclear factor kappa-light-chain-enhancer of activated B; p-Akt = phosphorylated protein kinase B; p-GSK3*β* = phosphorylated glycogen synthase kinase 3*β*; t-GSK3*β* = total glycogen synthase kinase 3*β*; t-Akt = total protein kinase B; TNF-*α* = tumour necrosis factor *α*.

Protein samples were separated on 10% or 15% polyacrylamide gels of 1.5 mm thickness. Stacking gel contained 0.5 M Trizma base (T1504, Sigma-Aldrich) at pH 6.8, 10% sodium dodecyl sulfate (SDS; L3771, Sigma-Aldrich), 30% acrylamide (161-0156, Bio-Rad, Hercules, CA), 10% ammonium persulfate solution (APS; A3678, Sigma-Aldrich), tetramethylenediamine (TEMED; T9281, Sigma-Aldrich), and dH_2_O. Separating gel contained 1.5 M Trizma base at pH 8.8, 10% SDS, 30% acrylamide, 10% APS, TEMED, and distilled H_2_O.

Samples were diluted in SDS buffer (10% *w*/*v* SDS, 10 mM dithiothreitol, 20% *v*/*v* glycerol, 0.2 M Tris-HCl at pH 6.8, 0.05% *w*/*v* Bromophenol blue (all Sigma-Aldrich)) and Milli-Q H_2_O. Protein samples were denatured at 60°C for 20 minutes. 20 *μ*g of protein was loaded per lane, and 5 *μ*L of precision plus western C (161-0376, Bio-Rad) was loaded into the first lane of each gel to use as a molecular ladder. The gels were run for 30 minutes at 60 V in SDS running buffer containing 25 mM Tris-HCl, 200 mM glycine (Sigma-Aldrich), and 0.1% *w*/*v* SDS. When the dye front had moved through the stacking gel, gels were run for a further 1-1.5 hours at 110 V. Gels were electrotransferred onto polyscreen polyvinylidene difluoride (PVDF) membranes (NEF100200, Perkin-Elmer, Waltham, MA) for 1.5 hours at 100 V at 4°C using transfer buffer containing 0.058% Trizma base, 10% SDS, 20% methanol, and 0.029% glycine. Following transfer, PVDF membranes were blocked with 30 mL of 0.5% skim milk solution containing Tris-buffered saline (TBST, Trizma base, NaCl at pH 7.5 and Tween20 (Sigma-Aldrich)) using the SNAP i.d. system (Merck Millipore). PVDF membranes were incubated with primary antibodies (Table 1) diluted in 0.5% skim milk/TBST overnight at 4°C on a blood tube roller.

The following day, membranes were washed with one litre of phosphate-buffered saline/Tween20 (PBST). Secondary antibody, goat anti-rabbit IgG (H+L)-conjugate (170-6515, Bio-Rad) was diluted in 0.5% skim milk/TBST solution, added to each membrane, and incubated at room temperature for 10 minutes. Membranes were then washed with one litre of PBST. Enhanced chemiluminescence (Merck Millipore) was added to the membranes and incubated at room temperature for one minute. Membranes were exposed to medical imaging film (28906839, GE Healthcare Life Sciences, Marlborough, MA). Film slides were scanned, and densitometry analysis was performed using ImageJ (https://imagej.nih.gov/ij/).

Few studies have reported western blot analyses using the D2-*mdx* mouse model. GAPDH is a standard western assay protein loading control that has also commonly been used in *mdx* mouse models [52]. Although mRNA expression did not vary (see below), protein expression of GAPDH was found to vary greatly between age-matched D2-*mdx* mice. Consequently, gels were run for Coomassie blue staining and normalisation to total protein content. Highly variable GAPDH expression in the D2-*mdx* mouse model was also recently reported by Kennedy and colleagues [53]. A recent study by Hildyard and others [54] in *mdx* mice showed that GAPDH gene expression exhibited a twofold decrease in dystrophic tissue highlighting GAPDH gene expression instability and marked muscle and *mdx*-specific differences.

After electrophoresis, separated proteins on acrylamide gels were placed in a clean container and rinsed three times for five minutes each with deionized water to remove excess SDS. 40 mL of EZBlue™ gel staining reagent (Coomassie blue G1041, Sigma-Aldrich) was added to each gel, and the gel was placed on a shaker and left to stain for one hour at room temperature. Following this, the gel was washed with deionized water every 10-15 minutes for four hours. The gel was then left on the shaker in deionized water overnight to destain. Images of the gel were taken the following day and analysed by densitometry.

### 2.8. RNA Analysis

Real-time quantitative polymerase chain reaction (RT-qPCR) analysis was performed using left ventricular myocardium (50 mg) from DBA/2 and D2-*mdx* mice frozen at necropsy to determine messenger RNA (mRNA) expression of several key genes. Total RNA extraction from TRI Reagent-treated tissue homogenates was performed as per manufacturer's instructions (Applied Biosystems, USA). RNA concentration and quality (Abs260/280 ratio between 1.8 and 2) of each sample were measured using the NanoDrop ND-1000 Spectrophotometer (Biolab, Canada). Extracted RNA samples were diluted to 100 ng/*μ*L and reverse transcribed to produce complementary deoxyribonucleic acid (cDNA) using the High-Capacity cDNA Reverse Transcription Kit (Applied Biosystems) and TaqMan assays listed in Table 2 were performed according to the manufacturer's protocol (Applied Biosystems). All reactions were run in triplicate with a 7900HT Fast Real-Timer PCR System (Applied Biosystems) with the thermocycler conditions: stage 1: 50°C for two minutes, 1 cycle; stage 2: 95°C for 10 min, 95°C for 15 seconds and 60°C for one minute, 40 cycles.

GAPDH = glyceraldehyde 3-phosphate dehydrogenase; PGC-1*α* = peroxisome proliferator-activated receptor-*γ* coactivator 1; IL-6 = interleukin 6; NF-*κ*B=nuclear factor ĸ-light-chain-enhancer of activated B cells; TNF-*α* = tumour necrosis factor *α*; Col1a1 = collagen type I *α* 1 chain; TLR4 = toll-like receptor 4.

Expression of mRNA was evaluated from the threshold cycle (CT) values. The CT value is the fractional PCR cycle at which the fluorescence signal passes the fixed threshold and reaches a level of detection that can be used to indicate the level of mRNA of the target gene. CT levels were analysed using the ∆CT method of relative quantification whereby ∆CT values are obtained by normalising each mRNA of interest to a reference mRNA (i.e., GAPDH) [55]. Relative fold change (FC) in gene expression was calculated as 2^-∆∆CT^, where FC = 1 means no change; FC > 1 = increase; FC < 1 = decrease in gene expression.

### 2.9. Statistical Analyses

All graphs and statistical analyses were completed using GraphPad Prism Software Version 9 (GraphPad Software Inc., La Jolla, CA). The Kolmogorov-Smirnov test was used to determine whether data was normally distributed, and statistical tests to contrast potential group differences were selected accordingly. For normally distributed data, one-way ANOVA, repeated measures two-way ANOVA, and Dunnett's multiple comparisons were used, comparing each group to the D2-*mdx* Vehicle group. Data are presented as mean ± standard deviation (SD). Where required, a Mann–Whitney *U* test was used, with data presented as individual values and group median.

## 3. Results

### 3.1. Skeletal and Respiratory Muscle in the D2-*mdx* Mouse

**Figure 1:**
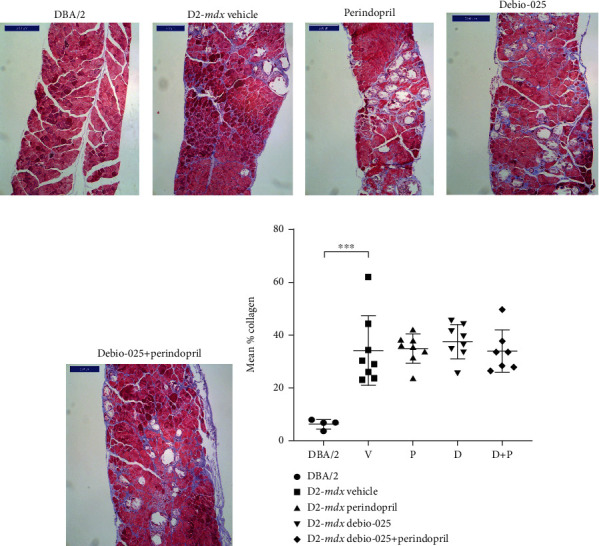
Fibrosis in diaphragms of DBA/2 and D2-*mdx* mice at 18 weeks. Representative transverse sections of diaphragms (Masson's trichrome stain, scale bar = 200 *μ*m) from (a) DBA/2 and D2-*mdx* treated to (b) Vehicle, (c) Perindopril, (d) Debio-025, and (e) Debio-025+Perindopril. Mean % collagen for each group (f). Data analysed by one-way ANOVA and Dunnett's multiple comparisons and presented as mean ± SD and individual values; ^∗∗∗^*p* < 0.001.

Male D2-*mdx* mice weighed less than age-matched DBA/2 mice at 15, 16, 17, and 18 weeks of age (*p* < 0.05; Table 3), and quadriceps weight was significantly lighter at 18 weeks (*p* = 0.0058; Table 4). Forelimb grip strength of the D2-*mdx* mice was weaker at 10 and 18 weeks compared to that of the DBA/2 mice (*p* = 0.004, *p* < 0.0001, respectively; Table 5). Diaphragms of the D2-*mdx* had a greater percentage of collagen compared to those of the DBA/2 mice at 18 weeks (*p* < 0.0001; Figure 1). No differences in any of these parameters were observed in the Perindopril, Debio-025, and Debio-025+Perindopril groups compared to the D2-*mdx* Vehicle group (Tables 3–5).

Data analysed by repeated measures two-way ANOVA and Dunnett's multiple comparison tests and presented as mean ± SD; ^∗^*p* < 0.05 vs. Vehicle D2-*mdx*.

Tibialis anterior (TA) and quadriceps (Qu) weights were normalised to tibia lengths. Tibial bone lengths were similar in all animals and did not differ between groups. Data analysed by one-way ANOVA and Dunnett's multiple comparisons and presented as mean ± SD; ^∗∗^*p* < 0.01 vs. Vehicle D2-mdx.

Forelimb grip strength is normalised to body weight. Data were analysed by repeated measures two-way ANOVA and Dunnett's multiple comparisons tests and presented as mean ± SD; ^∗∗^*p* < 0.01 and ^∗∗∗^*p* < 0.0001 vs. Vehicle D2-mdx.

### 3.2. Echocardiography

The structural and functional parameters of LV were measured at 10 and 18 weeks of age using echocardiography (Tables 6 and 7). No differences in fractional shortening and ejection fraction were observed between the DBA/2 and D2-*mdx* mice. Heart rate was greater in the DBA/2 mice compared to the D2-*mdx* mice at 18 weeks (*p* < 0.05). However, heart rates in all groups remained above 300 bpm and other functional parameters were not different between DBA/2 and D2-*mdx*. No differences were observed in LV structural or functional parameters in the treatment groups compared to the D2-*mdx* Vehicle group.

FS = fractional shortening; EF = ejection fraction; HR = heart rate; bpm = beats per minute. Data analysed by repeated measures two-way ANOVA and Dunnett's multiple comparisons tests and presented as mean ± SD; ^∗^*p* < 0.05 vs. D2-mdx Vehicle.

LV = left ventricle; LVED = LV end-diastolic; LVES = LV end-systolic; IVS = interventricular septum; PWT = posterior wall thickness. Data analysed by repeated measures two-way ANOVA and presented as mean ± SD.

### 3.3. Cardiac Mass

**Figure 2:**
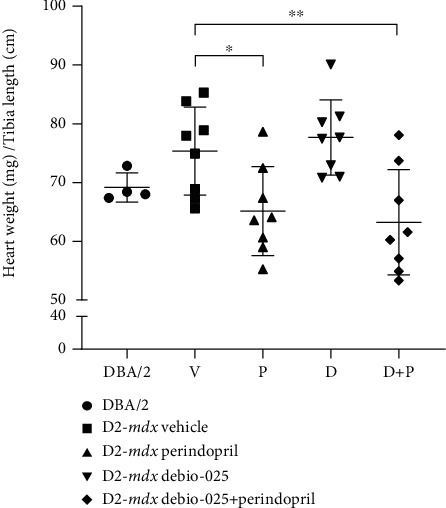
Heart weights (normalised to tibial length) from DBA/2 and D2-*mdx* mice at 18 weeks. Tibial bone lengths were similar in all animals and did not differ between groups. Data analysed by one-way ANOVA and Dunnett's multiple comparisons tests and presented as mean ± SD and individual values; ^∗^*p* < 0.05 and ^∗∗^*p* < 0.01.

No differences were observed in heart weights of the DBA/2 mice compared to those of the D2-*mdx* Vehicle mice at 18 weeks (Figure 2). Whole hearts from the Perindopril and Debio-025+Perindopril groups were lighter than those from the D2-*mdx* Vehicle mice (*p* = 0.03 and *p* = 0.009, respectively).

### 3.4. Cardiac Fibrosis

**Figure 3:**
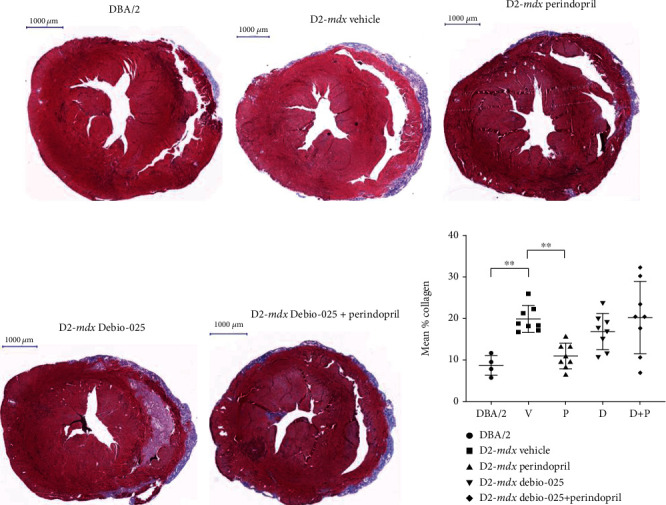
Cardiac fibrosis in DBA/2 and D2-*mdx* mice at 18 weeks. Representative sections of (a) DBA/2, (b) D2-*mdx* Vehicle, (c) D2-*mdx* Perindopril, (d) D2-*mdx* Debio-025, and (e) D2-*mdx* Debio-025+Perindopril mouse hearts stained with Masson's trichrome stain (scale = 1000 *μ*m). (f) Mean % collagen for DBA/2 and D2-*mdx* hearts. Data analysed by one-way ANOVA and Dunnett's multiple comparisons tests and presented as mean ± SD and individual values; ^∗∗^*p* < 0.01.

Transverse heart sections were assessed at 18 weeks of age using Masson's trichrome staining (Figures 3(a)–(e) All D2-*mdx* hearts featured an epicardial fibrotic calcinosis layer surrounding the right ventricle and extending beyond the edges of the LV. In contrast, while all the DBA/2 also had some RV epicardial calcinosis, this was very limited and restricted to the RV. The percentage of total area with fibrotic tissue was quantified using a digital segmentation method. Hearts of the D2-*mdx* mice had a greater percentage of collagen stain compared to those of the DBA/2 mice at 18 weeks (*p* = 0.004; Figure 3(f)), and *Col1a1* mRNA expression measured by RT-qPCR was greater in the D2-*mdx* hearts compared to the DBA/2 (FC = 1.89, *p* = 0.0008). Hearts from the D2-*mdx* Perindopril group had a lower percentage of collagen stain compared to those from the D2-*mdx* Vehicle group (*p* = 0.006; Figure 3(f)).

**Figure 4:**
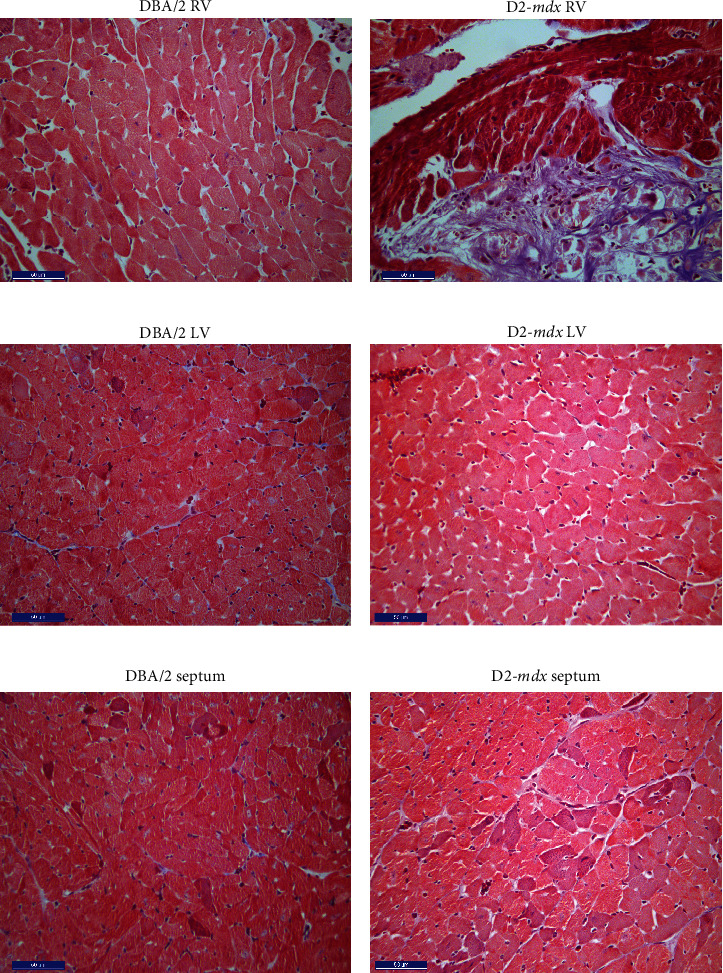
Cross-sectional images from the RV, LV, and septum of DBA/2 and D2-*mdx* hearts at 18 weeks. Representative sections of (a) DBA/2 RV, (b) D2-*mdx* RV, (c) DBA/2 LV, (d) D2-*mdx* LV, (e) DBA/2 septum, and (f) D2-*mdx* septum stained with Masson's trichrome (scale bar = 50 *μ*m).

**Figure 5:**
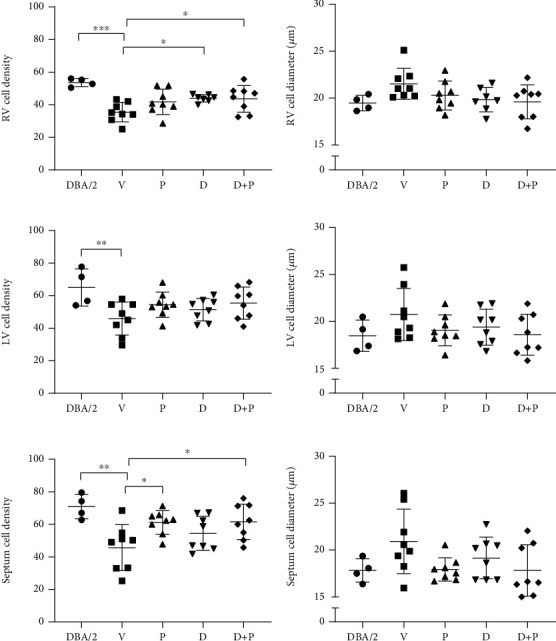
RV, LV, and septum cell density (number of cells/area) and diameter in DBA/2 and treated D2-*mdx* mice at 18 weeks. The number of cells within an area of 19,500 *μ*m^2^ (150 × 130 *μ*m) for (a) RV, (c) LV, and (e) septum and average cell diameter for (b) RV, (d) LV, and (f) septum. Values represent the average of five areas of size 150 × 130 *μ*m assessed for each tissue region. Data were analysed by nested one-way ANOVA and presented as mean ± SD and individual values; ^∗^*p* < 0.05, ^∗∗^*p* < 0.01, and ^∗∗∗^*p* < 0.001.

In order to determine potential changes in cardiomyocyte content and cell size, average cell density and cell diameter were measured in the RV, LV, and septum of DBA/2 and D2-*mdx* mice at 18 weeks (Figures 4(a)–4(e) Greater RV cell density was observed in the DBA/2-, Debio-025-, and Debio-025+Perindopril-treated groups compared to the D2-*mdx* Vehicle group (*p* = 0.0002, *p* = 0.045, and *p* = 0.048, respectively, Figure 5). Greater LV and septum cell density was observed in the DBA/2 mice compared to the D2-*mdx* mouse model (*p* = 0.006 and *p* = 0.002, respectively). Septum cell density was greater in the Perindopril and Debio-025+Perindopril groups compared to Vehicle (*p* = 0.024 and *p* = 0.02, respectively).

### 3.5. Inflammation and Proapoptotic Activity

**Figure 6:**
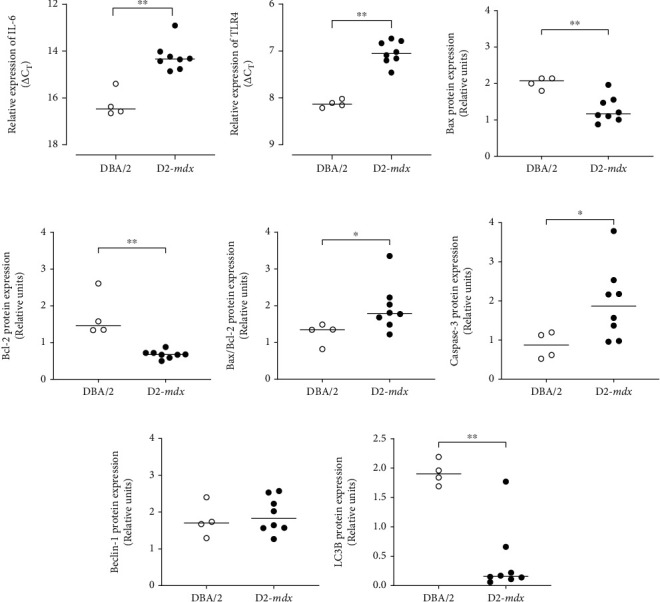
Relative expression of mRNA and proteins involved in inflammation, apoptosis, and autophagy in DBA/2 and D2-*mdx* hearts at 18 weeks. Expression of (a) IL-6 mRNA and (b) TLR4 mRNA obtained from RT-qPCR and presented as individual *Δ*CT values, where a lower *Δ*CT denotes greater mRNA expression. Relative protein expression of (c) Bax, (d) Bcl-2, (e) Bax/Bcl-2 ratio, (f) caspase-3, (g) Beclin-1, and (h) LC3B obtained from western immunoblots. Intact western blots are shown in Supplement 1 Data analysed by Mann–Whitney *U* test and presented as median and individual values; ^∗^*p* < 0.05 and ^∗∗^*p* < 0.01.

*IL-6* and *TLR4* mRNA expression levels were greater in the D2-*mdx* hearts compared to the DBA/2 (FC = 4.03, *p* = 0.006; FC = 2.13, *p* = 0.004, respectively; Figures 6(a) and 6(b) No significant differences were observed for *NF-κB* (FC = 1.0) and *TNF-α* (FC = 0.607) mRNA. Expression of Bax (proapoptotic marker) and Bcl-2 (antiapoptotic marker) proteins was lower in the D2-*mdx* hearts compared to the DBA/2 (*p* = 0.008 and *p* = 0.004, respectively; Figures 6(c) and 6(d)). Expression of the Bax/Bcl-2 ratio and caspase-3 was greater in the D2-*mdx* hearts compared to the DBA/2 (*p* = 0.049 and *p* = 0.049, respectively; Figures 6(e) and 6(f)). Lower levels of LC3B protein expression were observed in the D2-*mdx* hearts compared to the DBA/2 (*p* = 0.008; Figure 6(h)), but there was no difference in Beclin-1 expression. Compared to Vehicle, treatment with Debio-025, Perindopril, or their combination had no significant effect on relative expression of these inflammatory and apoptotic proteins (Table 8). See Supplement 1 for western assay blots.

Data presented as mean ± SD. Western blots are shown in Supplement 1.

### 3.6. Mitochondrial Perturbations

**Figure 7:**
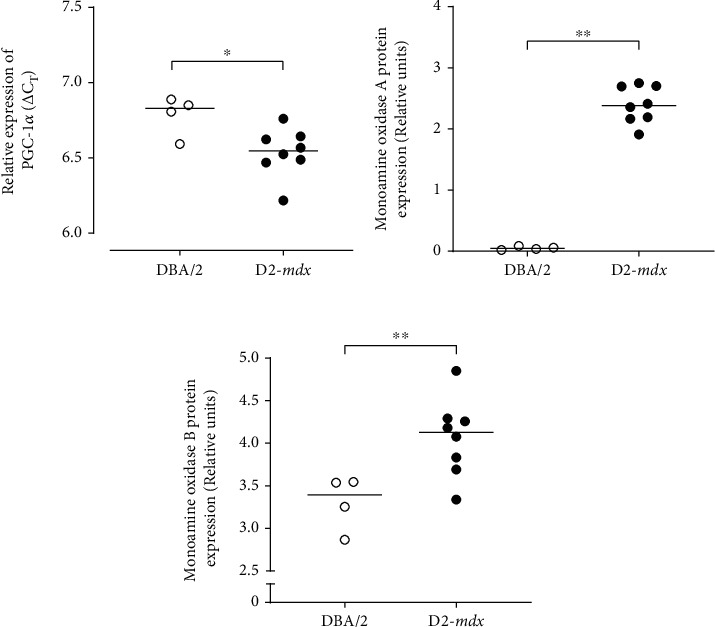
Relative expression pattern of PGC-1*α* gene and protein expression of markers of mitochondrial ROS signalling from monoamine oxidase in DBA/2 and D2-*mdx* hearts at 18 weeks. (a) Relative expression of PGC-1*α* obtained from RT-qPCR and presented as individual *Δ*CT values, where a lower *Δ*CT denotes greater mRNA expression. (b, c) Relative protein expression of monoamine oxidase A and monoamine oxidase B obtained from western immunoblots. Data analysed by Mann–Whitney *U* test and presented as median and individual values; ^∗^*p* < 0.05 and ^∗∗^*p* < 0.01.

**Figure 8:**
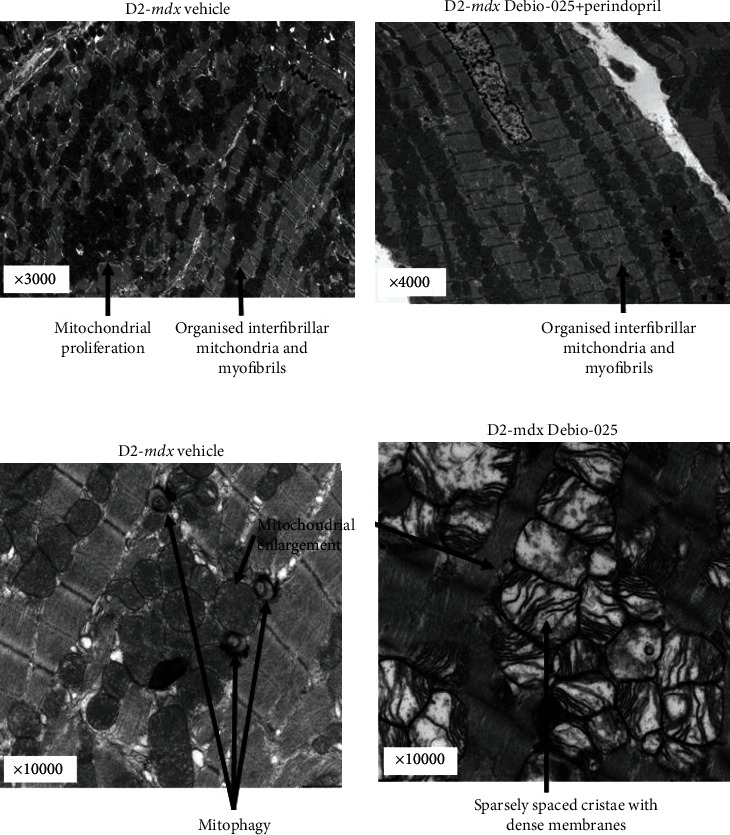
Representative electron micrographs of LV myocardial tissue from D2-*mdx* mice at 18 weeks. (a) An example from the D2-*mdx* Vehicle group that shows areas of randomly distributed mitochondria between poorly organised mitochondria, mitochondrial proliferation, and also some areas that are preserved (3000x mag). (b) Highly preserved regions with evenly sized and distributed mitochondria in the D2-*mdx* Debio-025+Perindopril group (4000x mag). (c) Examples of mitophagy and mitochondrial swelling in the D2-*mdx* Vehicle group (10,000x mag). (d) Examples of enlarged mitochondria with thick membranes and sparse cristae in the D2-*mdx* Debio-025 group (10,000x mag).

Relative expression of the *PGC-1α* gene and monoamine oxidase A and monoamine oxidase B proteins (MAO-A, MAO-B) was greater in the hearts of the D2-*mdx* mice compared to the DBA/2 mice at 18 weeks (FC = 1.19, *p* = 0.028; *p* = 0.004; *p* = 0.016, respectively; Figure 7). No effect of treatment with Debio-025 (FC = 0.999, *p* = 0.999), Perindopril (FC = 1.19, *p* = 0.079), or their combination (FC = 1.19, *p* = 0.145) was evident for *PGC-1α* or MAO-A and MAO-B proteins (Table 8). Representative electron micrographs of the LV from the D2-*mdx* Vehicle group were comparable to those from the Perindopril, Debio-025, and Perindopril+Debio-025 treatment groups (Figure 8). Despite heterogeneous areas of disorganisation and ultrastructural variation, all treatment groups also displayed discrete areas of evenly distributed interfibrillar mitochondria between organised myofibrils. All D2-*mdx* group mitochondria also featured distinct areas of mitochondrial proliferation, mitochondrial swelling, loss of cristae, and mitophagy. Although the electron micrograph assessment did not permit quantitative analysis, the mitochondrial and myofibril morphology indicates that these adaptive perturbations within anaesthetized D2-*mdx* mice were adequate to sustain resting LV function.

## 4. Discussion

In the current era of improved respiratory management in DMD patients, cardiomyopathy progressing to heart failure is the main cause of death [11]. In this study, the D2-*mdx* mouse model was characterized and tested to determine whether the ACE inhibitor Perindopril, already in clinical use, and Debio-025, a specific mPTP blocker with a human safety profile, either alone or in combination could have an impact on the extent of cardiomyopathy. The main findings were as follows: (1) at 18 weeks of age, compared to DBA/2 mice, D2-*mdx* mice had a severe cardiac pathology characterized by fewer myocytes (less cell density), larger myocytes (hypertrophy), and intense epicardial fibrosis, most prominently in the RV; (2) resting LV function was comparable to age-matched DBA/2 mice at 18 weeks; (3) following a six-week drug treatment period from 12 to 18 weeks, Perindopril marginally reduced total heart weight and collagen content at 18 weeks of age in D2-*mdx* mice. However, Perindopril, Debio-025, and their combination had no significant impact on LV function, and treatment with both drugs did not further limit the extent of fibrosis. Notably, the indirect echocardiographic measure of LV mass was not altered by Perindopril treatment.

The lower forelimb force, body, and muscle weights in comparison to the DBA/2 animals are consistent with other reports of an atrophic phenotype in D2-*mdx* mice [3, 25, 40–43]. Despite the overt epicardial pathology in D2-*mdx* mice at 18 weeks, it appears that by this age these mice maintain sufficient adaption to sustain normal resting LV function. Thus, not surprisingly, Debio-025, Perindopril, and their combination had no discernible impact on LV performance. However, the decreased heart rate in the D2-*mdx* mice compared to the DBA/2 at 18 weeks linked with relatively similar LV dimensions and ejection fraction, but lower heart weight suggests potential for lower cardiac output. If the LV length in D2-*mdx* mice is shorter than that in DBA/2 mice, then a more globular LV geometric shape may lead to overestimated ejection fraction because the present cardiac ultrasound analysis is based on m-mode assumptions of similar LV diameter and length.

An early study reported marked cardiac dysfunction in the D2-*mdx* mice compared to DBA/2 [40]. In contrast, our study and other studies to date have not been able to successfully reproduce these findings. Kennedy and colleagues [53] assessed cardiac function at 21 weeks using cardiac MRI and found that, compared to DBA/2 mice, D2-*mdx* mice showed elevated LV and RV EF. Yet, at 28 weeks, these differences were no longer apparent. Vohra and colleagues [41] used cardiac MRI and were not able to detect LV dysfunction compared to DBA/2 mice at 28 weeks. Hughes and colleagues [25] used echocardiography and detected no overt cardiac dysfunction at four weeks, and Hakim and colleagues [43] used a cardiac catheter assay and also found no differences between the D2-*mdx* and DBA/2 mice at six months, also reporting high EF values. However, they did detect characteristic ECG changes such as increased QRS duration and QT interval in the D2-*mdx* mice compared to the DBA/2. Our findings at 10 and 18 weeks indicate that there are no major differences in LV function.

### 4.1. Fibrosis and Calcinosis

Dystrophic myocardial calcinosis has been reported for several mouse strains including DBA/2 mice [56], which is consequent to inflammation, necrosis, fibrosis, and calcium mineralisation and is also linked to *dyscalc1* expression in D2-*mdx* mice [48]. The severe epicardial fibrosis measured in the D2-*mdx* mice prominently features calcinosis in a thick fibrotic outer layer surrounding the RV. This result is consistent with Kennedy and colleagues and van Putten and colleagues who also reported an increase in myocardial fibrosis at similar ages (10 and 21 weeks, respectively), as well as prominent necrosis and calcification [42, 53]. Hughes and colleagues [25] reported no differences in the degree of fibrosis between the D2-*mdx* and DBA/2 mice at four weeks of age, indicating severe myocardial fibrosis is likely to occur between four and 10 weeks of age. The present study is the first to examine average myocyte density and diameter in the RV, LV, and septum of DBA/2 and D2-*mdx* mice. Although Hughes et al. [25] did assess LV cardiomyocyte size, no myofibril atrophy or hypertrophy was detected in D2-*mdx* mice at four weeks.

In support of our trichrome stain histology results, we also observed elevated expression of *Col1a1* in the hearts of D2-*mdx* mice compared to DBA/2 mice at 18 weeks. This result supports the findings of van Putten et al. [42] who reported similar elevated expression at 10 weeks of age. *Col1a1* is a gene involved in collagen synthesis and is likely to be elevated in these animals due to the identified polymorphism in the Ltpb4 gene found in their DBA/2 background [44]. This polymorphism results in upregulated signalling of the profibrotic TGF-*β* pathway, increasing Col1a1 gene expression, a major component of the extracellular matrix [56]. TGF-*β* is a potent profibrogenic factor that, in conjunction with downstream SMAD signalling, stimulates fibroblasts to produce extracellular matrix proteins such as collagen and fibronectin and is activated in response to stress or injury [57]. Contrary to our findings and previous literature comparing the D2-*mdx* and DBA/2 mice, Hakim and colleagues reported pathological changes present in the DBA/2 wild-type mice as well as the D2-*mdx*^43^. Specifically, they observed epicardial fibrotic and calcified areas that were located on the surface of the RV, as well as regions of fibrosis throughout the septum and LV, comparable to the pathology detected in the D2-*mdx* mice [43]. In our study, we observed only a small degree of fibrosis in the DBA/2 hearts. Variation in these results may be explained by the fact that previous reports have found that only approximately half the mice on the DBA background show cardiac calcification [56]. It is also possible that the polymorphism found in the background DBA/2 of these animals contributed to a greater propensity for fibrosis in the D2-*mdx* mice. However, a limitation of our study was that, due to division of the same hearts for other measures, histological assessment was conducted only at a single cut plane, thus preventing detailed quantitative analyses through each entire heart. Given the limitations on available animals and the unique epicardial RV-focused fibrosis and calcinosis in this study that is not evident in patients with DMD, the present data and animal model are limited with regard to direct experimental support of the current clinical use of Perindopril where it is primarily used to reduce myocardial fibrosis to delay the onset of cardiomyopathy in DMD patients [58].

### 4.2. Adaptive Cell Signalling Processes

The expression of genes and proteins involved in apoptosis, autophagy, inflammation, and mitochondrial ROS signalling in the myocardium of D2-*mdx* animals has not previously been studied in detail. We found that expression of Bax/Bcl-2 and caspase-3 was significantly increased in the D2-*mdx* mouse hearts compared to the DBA/2, suggesting greater likelihood of apoptosis in D2-*mdx* high ratios of Bax (proapoptotic factor) to Bcl-2 (antiapoptotic factor) are indicative of propensity for apoptosis in mitochondrial-mediated apoptotic pathways that involve sustained opening of the mPTP and therefore release cytochrome c from the mitochondria [59]. The release of mitochondrial cytochrome c associates with apoptotic protease-activating factor and procaspase-9 to form the apoptosome, which then activates caspase-9 and caspase-3 [59, 60].

Contrary to previous reports, we found no differences in Akt or GSK3*β* between D2-*mdx* and DBA/2 hearts. Recent studies have suggested a role for these two proteins in the development of cardiac hypertrophy through Akt activation and GSK3*β* inhibition [61]. GSK3*β* inhibition can occur via stimuli that activate phosphatidylinositol 3-kinase (PI3K), which acts via Akt, an upstream regulator of GSK3*β*, and by activation of the Wnt signalling pathway [62, 63]. We were however unable to confirm Kennedy and colleagues' report of increased phosphorylated GSK3*β* relative to total GSK3*β* expression in the D2-*mdx* hearts compared to DBA/2 at 21 weeks [53].

As a measure of cardiac hypertrophy, heart weight was normalised to tibia length, and although there was a trend for greater heart weight in the D2-*mdx* mice, there was no significant difference compared to the DBA/2. Although we detected larger, but fewer cardiomyocytes, these histological measures were limited to a single plane and not a systematic series of sections through the entire heart; thus, a more detailed quantitative histological study is required.

We also assessed markers of autophagy, which is an important protective mechanism that promotes degradation and recycling of cellular components and occurs in normal heart maintenance [64]. A recent study by Fiacco and colleagues [65] showed that activation of autophagy was impaired during the late stages of DMD progression and that this correlated with a decline in muscle regeneration and the accumulation of fibrotic tissue in *mdx* mice. Our current finding suggests that at least LC3B-related activity may be impeded in D2-mdx hearts. In addition, the previous reports of chronic inflammation associated with DMD and various *mdx* models [66, 67] were supported by our findings of greater cardiac TLR4 and IL-6 gene expression in D2-*mdx* compared to DBA/2 mice. However, these inflammatory and autophagy signalling markers were not responsive to our current drug treatment regimes.

### 4.3. Mitochondrial Adaptations

We have identified unique and important evidence for mitochondrial adaptations and increased ROS in the hearts of the D2-*mdx* mice, as indicated by elevated PGC-1*α*, MAO-A, and MAO-B. MAO-A and MAO-B are two isoenzymes located on the outer mitochondrial membrane and play a role in oxidative deamination of monoamines (from neurotransmitters and diet) producing aldehydes, ammonia, and H_2_O_2_, with potential to contribute to ROS and hydroxyl radical accumulation in the heart [68, 69]. Increased MAO results in production of H_2_O_2_ that can contribute to important adaptations via intracellular signalling of ERK1/2, NFAT3/4, and matrix metalloproteases, which in turn promote cell hypertrophy, fibroblast proliferation, and extracellular matrix remodelling [70]. Subsequent maladaptation causes mitochondrial perturbation, mPTP opening, apoptosis, and ultimately cardiac dysfunction and further remodelling [70]. Increased expression of PGC-1*α* in the D2-*mdx* mice suggests that mitochondrial biogenesis and mitochondrial proliferation may be upregulated as an adaptation to maintain viable mitochondria and energy production. Recent studies have found that upregulating PGC-1*α* in *mdx* mice improves myofibril recovery following injury and reduces degeneration [71]. Our results extend the findings of Hughes and colleagues who identified mitochondrial dysfunction at four weeks of age in D2-*mdx* mice [25]. Their work identified complex I-supported H_2_O_2_ emission and impaired mitochondrial respiration as preceding steps before any signs of cardiac dysfunction contrast to the BL10-*mdx* mouse model where early mitochondrial dysfunction in dystrophic hearts remains undetected due to the extensive muscle regeneration experienced by these animals [72]. Hughes and colleagues [25] have proposed that early mitochondrial dysfunction is easier to detect because the D2-*mdx* mice have abnormal regenerative capacity in skeletal muscle.

Representative electron micrographs of LV from the D2-*mdx* showed extensive compensatory mitochondrial proliferation and resultant disrupted myofibril organisation. Within the zones of mitochondrial proliferation, there was also evidence of varying stages of mitochondrial swelling and mitophagy. Although a systematic quantitative electron micrograph study was not possible, compared to Vehicle controls, drug-treated hearts featured extensive discrete areas of mitochondrial proliferation and dense cristae and areas of mitochondrial abundance amongst ordered myofibrils. Generally, all D2-*mdx* showed heterogeneous areas of disorganisation in contrast to discrete areas showing evenly distributed interfibrillar mitochondria between organised myofibrils. These organised areas are congruent with capacity for the normal resting LV function measured at 18 weeks.

### 4.4. Study Limitations

A limitation of our study is that cardiac ultrasound measures were made in anaesthetized animals with low resting heart rates. Increasing demand for cardiac output by workload due to exercise metabolism and increased heart rate may have evoked functional limitations in the LV. The present study was also limited due to the inability to obtain RV ultrasound imaging of sufficient quality for quantitative assessment of function in D2-*mdx* mice. Imaging of the RV in mice *in situ* is challenging due to interference from the sternum which affects accurate positioning of the ultrasound probe. Sternum artefact can also obscure portions of the heart, particularly the endocardial borders, making it difficult to assess RV function. Yet from our morphology sections, the extensive RV fibrosis in these mice suggests potential for RV dysfunction; thus, future studies require direct RV investigation with magnetic resonance imaging or pressure catheters in D2-*mdx* mice.

Another limitation of our study is that we were not able to initiate treatments at a younger age (i.e., at weaning) due to the commercial purchase and overseas shipment of the mice. Although no LV dysfunction was evident at 10 weeks of age, early progression of fibrosis may have limited potential for drug treatment impact on fibrosis. Debio-025 has been shown to be most effective when used in a preventative manner, prior to myofibril loss and replacement with fibrotic tissue [24, 37]. Thus, future study designs need to include capacity for treatment before the onset of fibrosis, i.e., *in utero*, at birth, or postweaning, and at later more advanced phases of adverse muscle remodelling. Another important study limitation was the use of DBA/2 mice as the comparator control group, due to the variability and propensity for early onset fibrosis and calcinosis in these mice; future studies of D2-*mdx* should concurrently include additional comparator control groups with other backgrounds.

## 5. Conclusions

D2-*mdx* mice displayed a severe diaphragm myopathy characterized by large regions devoid of myocytes and collagen replacement, consistent with other studies [40, 42] and DMD patients [73]. Treatment with Perindopril, Debio-025, or in combination did not reduce diaphragm collagen content compared to the Vehicle group. Similar to DMD patients [74, 75], we confirm that D2-*mdx* mice display severe fibrosis in advance of LV dysfunction, though predominantly in the RV. In contrast to DMD patients with early subendocardial and inferobasal LV fibrosis [75], D2-*mdx* mice had severe fibrosis predominantly in the RV, and there was no overt LV dysfunction in D2-*mdx* mice compared to age-matched DBA/2. Given the extensive respiratory diaphragm and RV fibrosis, it is likely that D2-*mdx* mice may have respiratory and RV dysfunction, which should be directly investigated in future studies.

We have identified novel characteristics of the D2-*mdx* mice that have not previously been reported, including reduced myocyte number, myocyte hypertrophy, augmented mitochondrial MAO and *PGC1α* expression, and increased myocardial fibrosis, most prominently in the RV. Thus, at 18 weeks of age, the D2-*mdx* mice appear sufficiently adapted to maintain resting LV function. Chronic Perindopril and Debio-025 treatment had no impact on fibrosis and myocyte disruption. Our results suggest that the adaptations evident in the D2-*mdx* mouse model have cardiomyopathy features which are only partly in common with those seen in patients with DMD, and thus, further detailed longitudinal and molecular investigations of this mouse model are required.

## Figures and Tables

**Table 1:** Antibody details for western immunoblotting.

Marker	Catalogue number	Company	Molecular weight (kDa)	Source	Dilution
Bax	2772	Cell Signalling, USA	20	Rabbit	1/1000
Bcl-2	2876	Cell Signalling, USA	28	Rabbit	1/1000
Beclin-1	3495	Cell Signalling, USA	60	Rabbit	1/500
Caspase-3	9665	Cell Signalling, USA	35	Rabbit	1/500
GAPDH	5174	Cell Signalling, USA	37	Rabbit	1/50,000
LC3B	3868	Cell Signalling, USA	16	Rabbit	1/1000
MAO-A	ab126751	Abcam, UK	60	Rabbit	1/1000
MAO-B	ab137778	Abcam, UK	60	Rabbit	1/1500
NF-*κ*B	12540	Cell Signalling, USA	50	Rabbit	1/500
p62	5114	Cell Signalling, USA	62	Rabbit	1/1000
p-Akt	4060	Cell Signalling, USA	60	Rabbit	1/1000
p-GSK3*β*	5558	Cell Signalling, USA	46	Rabbit	1/1000
t-GSK3*β*	9315	Cell Signalling, USA	46	Rabbit	1/1000
t-Akt	4691	Cell Signalling, USA	60	Rabbit	1/1000
TNF-*α*	11948	Cell Signalling, USA	28	Rabbit	1/500

**Table 2:** Primers for RT-qPCR.

Primer	Catalogue number	Target species	Company
GAPDH	4352932E	Mouse	Applied Biosystems
PGC-1*α*	Mm01208835_m1	Mouse	Applied Biosystems
IL-6	Mm00446190_m1	Mouse	Applied Biosystems
NF-*κ*B	Mm00482418_m1	Mouse	Applied Biosystems
TNF-*α*	Mm00443258_m1	Mouse	Applied Biosystems
Col1a1	Mm00801666_g1	Mouse	Applied Biosystems
TLR4	Mm00445273_m1	Mouse	Applied Biosystems

**Table 3:** Body weights of DBA/2 and D2-*mdx* mice from 10 to 18 weeks.

	Body weight (g)				
	DBA/2	D2-*mdx*			
		Vehicle	Perindopril	Debio-025	Debio-025+Perindopril
10 weeks	25.6 ± 2.6	21.8 ± 1.3	21.8 ± 1.3	21.7 ± 2.6	20.7 ± 2.4
11 weeks	24.8 ± 1.6	22.7 ± 1.7	22.7 ± 1.4	22.6 ± 2.2	21.4 ± 2.4
12 weeks	25.3 ± 1.8	22.9 ± 2.0	23.0 ± 1.2	22.8 ± 2.3	21.6 ± 2.1
13 weeks	25.4 ± 1.9	23.5 ± 1.8	23.4 ± 1.5	23.3 ± 2.2	21.9 ± 2.4
14 weeks	25.8 ± 1.5	23.9 ± 1.8	23.7 ± 1.0	23.9 ± 2.4	22.0 ± 2.7
15 weeks	27.2 ± 1.7^∗^	23.7 ± 1.5	23.6 ± 1.2	23.5 ± 2.4	21.9 ± 2.2
16 weeks	28.2 ± 2.1^∗^	23.9 ± 2.1	24.1 ± 1.3	23.9 ± 2.4	22.2 ± 2.2
17 weeks	28.3 ± 1.3^∗^	24.7 ± 1.7	24.7 ± 1.2	24.3 ± 2.6	23.1 ± 1.5
18 weeks	29.0 ± 1.7^∗^	24.9 ± 2.1	24.6 ± 1.3	23.8 ± 2.2	23.1 ± 1.2

**Table 4:** Tibialis anterior and quadriceps weights of DBA/2 and D2-*mdx* mice at 18 weeks.

	DBA/2	D2-*mdx*			
		Vehicle	Perindopril	Debio-025	Debio-025+Perindopril
TA (mg/cm)	19.8 ± 1.4	17.3 ± 2.9	16.5 ± 3.7	18.4 ± 2.7	13.7 ± 1.9
Qu (mg/cm)	56.1 ± 12.0^∗∗^	42.1 ± 5.0	38.5 ± 7.9	41.1 ± 4.4	38.9 ± 5.0

**Table 5:** Forelimb grip strength of DBA/2 and D2-*mdx* mice at 10 and 18 weeks.

	Forelimb grip strength normalised to body weight (N/g)				
	DBA/2	D2-*mdx*			
		Vehicle	Perindopril	Debio-025	Debio-025+Perindopril
10 weeks	0.03 ± 0.01^∗∗^	0.01 ± 0.003	0.01 ± 0.004	0.01 ± 0.003	0.01 ± 0.004
18 weeks	0.04 ± 0.004^∗∗∗^	0.02 ± 0.008	0.02 ± 0.005	0.025 ± 0.007	0.03 ± 0.005

**Table 6:** LV functional parameters in DBA/2 and D2-*mdx* mice at 10 and 18 weeks.

		DBA/2	D2-*mdx*			
			Vehicle	Perindopril	Debio-025	Debio-025+Perindopril
FS (%)	10 weeks	38 ± 9.50	39.5 ± 7.4	41.3 ± 6.7	33.4 ± 7.8	38.5 ± 5.9
	18 weeks	46.8 ± 9.4	40.8 ± 6.6	37.6 ± 9.2	39.9 ± 7.2	31.4 ± 8.4
EF (%)	10 weeks	68.6 ± 12.0	70.6 ± 8.8	72.7 ± 8.4	62.6 ± 11.4	69.6 ± 7.6
	18 weeks	78.2 ± 9.0	72.1 ± 8.3	67.4 ± 11.5	71.1 ± 9.0	59.0 ± 11.9
HR (bpm)	10 weeks	415.3 ± 50.3	363.5 ± 42.2	378.3 ± 53.3	342.3 ± 41.4	315.0 ± 43.6
	18 weeks	442.8 ± 58.5^∗^	348.0 ± 61.7	317.8 ± 56.5	309.8 ± 27.4	341.0 ± 63.1

**Table 7:** LV structural parameters in DBA/2 and D2-*mdx* mice at 10 and 18 weeks.

		DBA/2	D2-*mdx*			
			Vehicle	Perindopril	Debio-025	Debio-025+Perindopril
LVEDd (mm)	10 weeks	3.7 ± 0.2	3.3 ± 0.3	3.4 ± 0.5	3.5 ± 0.3	3.3 ± 0.4
	18 weeks	3.5 ± 0.2	3.5 ± 0.5	3.6 ± 0.4	3.4 ± 0.4	3.7 ± 0.4
LVESd (mm)	10 weeks	2.3 ± 0.4	2.0 ± 0.4	2.0 ± 0.4	2.3 ± 0.4	2.1 ± 0.4
	18 weeks	1.9 ± 0.4	2.1 ± 0.5	2.4 ± 0.7	2.1 ± 0.3	2.6 ± 0.5
IVSd (diastole) (mm)	10 weeks	0.5 ± 0.1	0.6 ± 0.1	0.6 ± 0.1	0.6 ± 0.1	0.6 ± 0.1
	18 weeks	0.7 ± 0.1	0.7 ± 0.1	0.7 ± 0.2	0.8 ± 0.2	0.8 ± 0.2
IVSd (systole) (mm)	10 weeks	0.8 ± 0.2	1.0 ± 0.3	1.1 ± 0.1	0.9 ± 0.2	1.0 ± 0.2
	18 weeks	1.2 ± 0.1	1.2 ± 0.2	1.2 ± 0.2	1.2 ± 0.2	1.2 ± 0.2
PWT (diastole) (mm)	10 weeks	0.5 ± 0.1	0.7 ± 0.2	0.6 ± 0.1	0.6 ± 0.2	0.7 ± 0.1
	18 weeks	0.8 ± 0.2	0.8 ± 0.2	0.8 ± 0.1	0.7 ± 0.2	0.8 ± 0.2
PWT (systole) (mm)	10 weeks	1.0 ± 0.2	1.0 ± 0.3	0.9 ± 0.2	0.9 ± 0.2	0.9 ± 0.2
	18 weeks	1.1 ± 0.1	1.1 ± 0.3	1.2 ± 0.1	1.0 ± 0.2	1.0 ± 0.3
LV mass (mg)	10 weeks	59.0 ± 11.6	63.6 ± 13.9	63.3 ± 15.2	69.0 ± 21.4	67.2 ± 15.3
	18 weeks	82.4 ± 21.2	85.3 ± 20.6	90.2 ± 10.9	85.4 ± 25.4	100.1 ± 28.1

**Table 8:** Summary of relative protein expression from western blot assays in treated D2-*mdx* hearts.

	Vehicle	Perindopril	Debio-025	Debio-025+Perindopril
Bax	2.23 ± 0.52	2.03 ± 0.47	2.56 ± 1.03	2.67 ± 0.63
Bcl-2	1.19 ± 0.26	1.34 ± 0.33	1.12 ± 0.30	1.18 ± 0.31
Bax/Bcl-2	1.95 ± 0.65	1.61 ± 0.54	2.33 ± 0.68	2.10 ± 0.75
Caspase-3	3.35 ± 1.58	3.0 ± 1.59	3.52 ± 0.97	4.10 ± 1.11
Beclin-1	3.37 ± 0.87	3.0 ± 0.54	3.10 ± 0.69	2.97 ± 0.79
MAO-A	4.18 ± 0.54	4.36 ± 1.06	4.07 ± 0.90	4.18 ± 1.44
MAO-B	7.09 ± 0.70	7.26 ± 0.76	6.65 ± 0.49	6.48 ± 0.51
NF-*κ*B	0.38 ± 0.19	0.44 ± 0.34	0.63 ± 0.62	0.63 ± 0.66
TNF-*α*	1.16 ± 0.84	0.47 ± 0.54	3.07 ± 1.78	0.72 ± 0.61

## Data Availability

Data can be found in the manuscript and supplementary data file.

## Supplementary Materials

Supplementary Materials Western blot protein expression assays (Supplement 1).
