# Supplementary material for: Preserved Left Ventricular Function despite Myocardial Fibrosis and Myopathy in the Dystrophin-Deficient D2.B10-Dmdmdx/J Mouse
Source: Oxid Med Cell Longev. 2022 Mar 16;2022:5362115. doi: 10.1155/2022/5362115 (PMC8942668; doi:10.1155/2022/5362115)

Appendix A. Western immunoblots for Bax (20kDa) in DBA/2 and treated D2-*mdx* hearts.
V=Vehicle, P=Perindopril, D=Debio-025, D+P=Debio-025+Perindopril

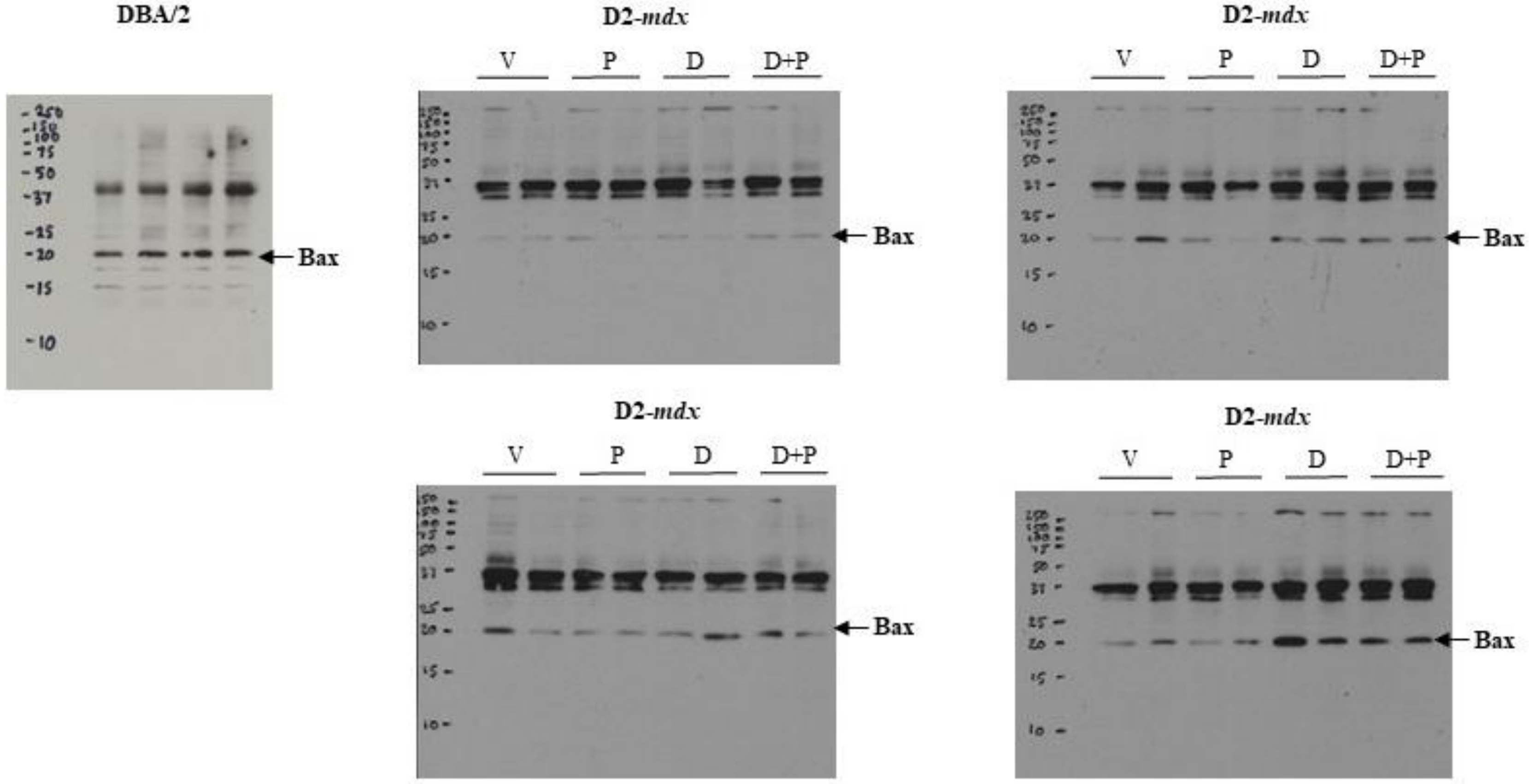

Appendix B. Western immunoblots for Bcl-2 (28kDa) in DBA/2 and treated D2-*mdx* hearts.
V=Vehicle, P=Perindopril, D=Debio-025, D+P=Debio-025+Perindopril

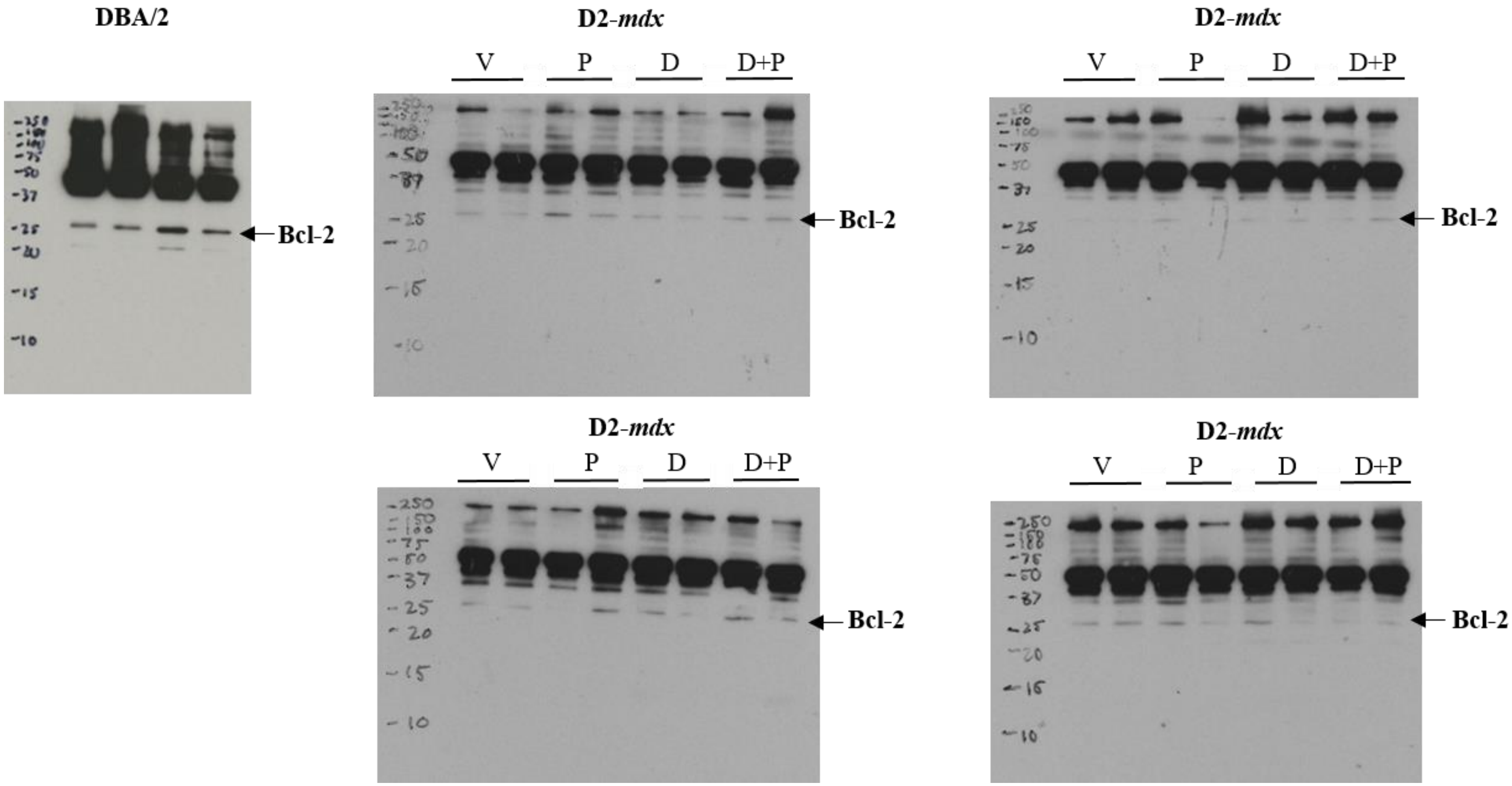

Appendix C. Western immunoblots for Caspase-3 (35kDa; uncleaved) in DBA/2 and treated D2-*mdx* hearts.
V=Vehicle, P=Perindopril, D=Debio-025, D+P=Debio-025+Perindopril

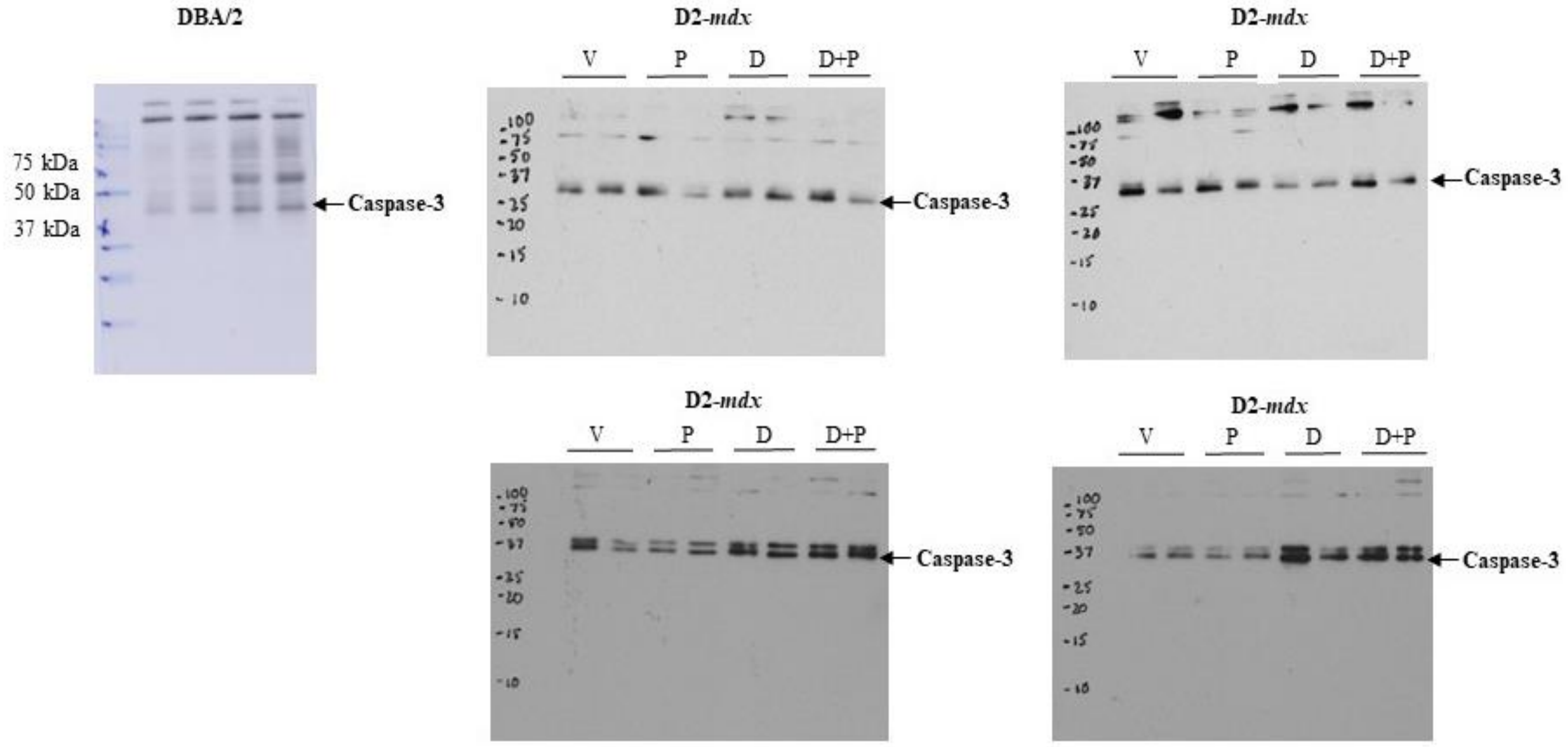

Appendix D. Western immunoblots for t-Akt (60kDa) and p-Akt (60kDa) in DBA/2 and D2-*mdx* hearts.

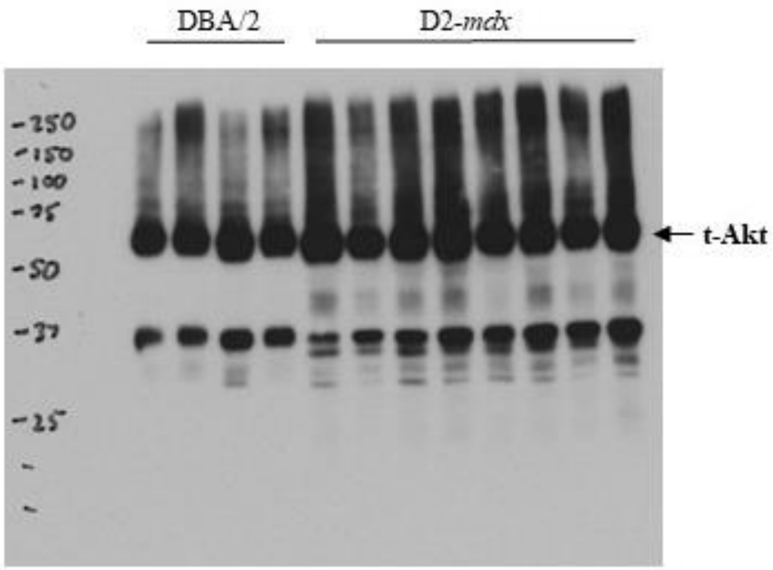

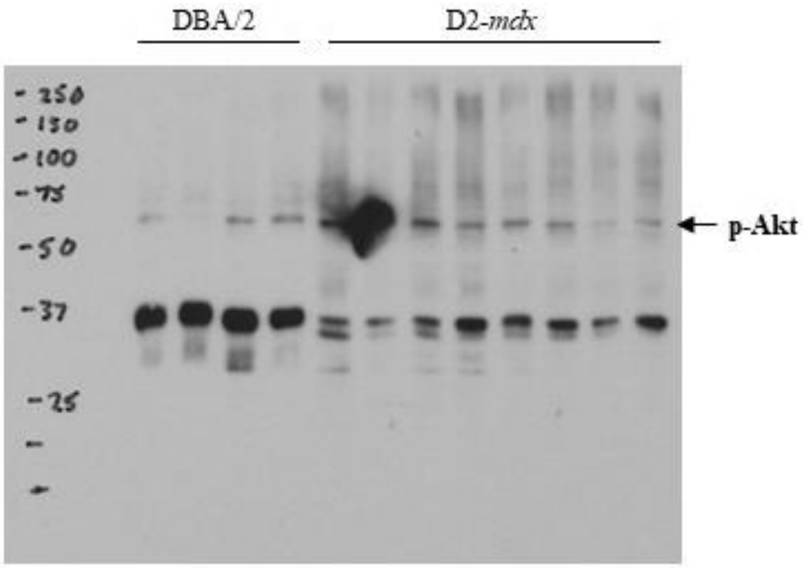

Appendix E. Western immunoblots for t-GSK3β (46kDa) and p-GSK3β (46kDa) in DBA/2 and D2-*mdx* hearts.

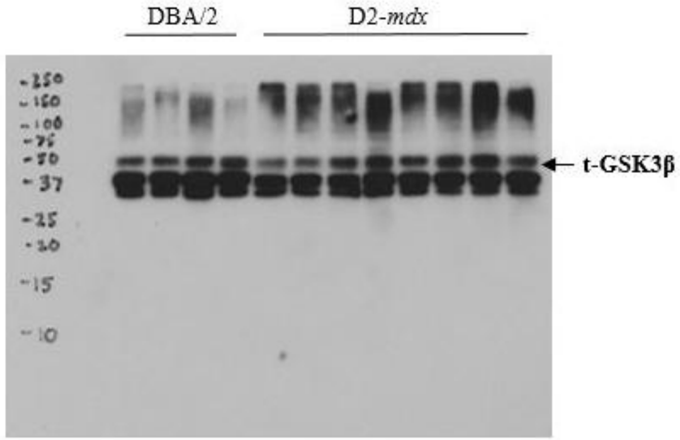

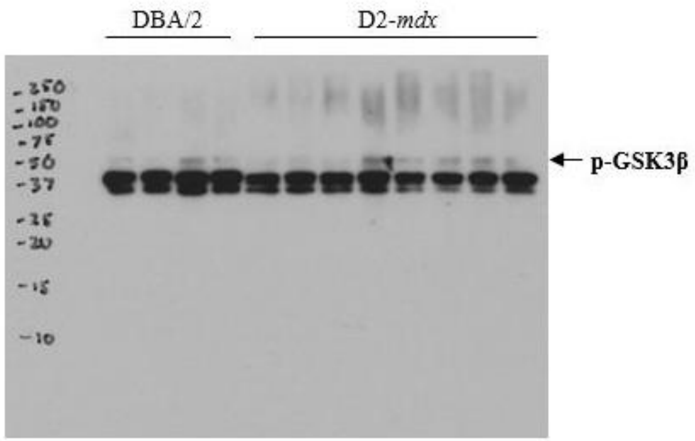

Appendix F. Western immunoblots for Beclin-1 (60kDa) in DBA/2 and treated D2-*mdx* hearts.
V=Vehicle, P=Perindopril, D=Debio-025, D+P=Debio-025+Perindopril

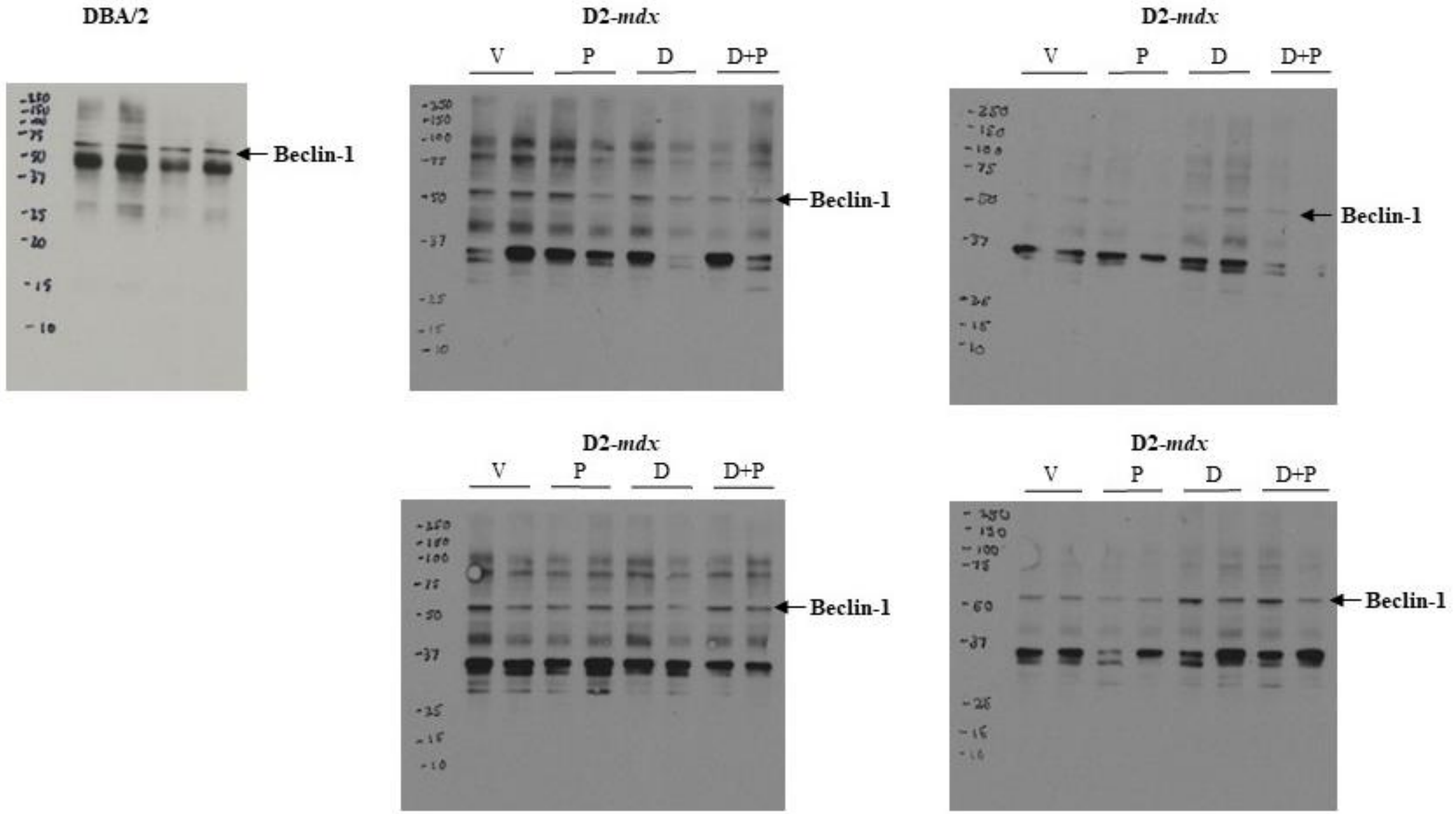

Appendix G. Western immunoblots for LC3B (16kDa) in DBA/2 and treated D2-*mdx* hearts.
V=Vehicle, P=Perindopril, D=Debio-025, D+P=Debio-025+Perindopril

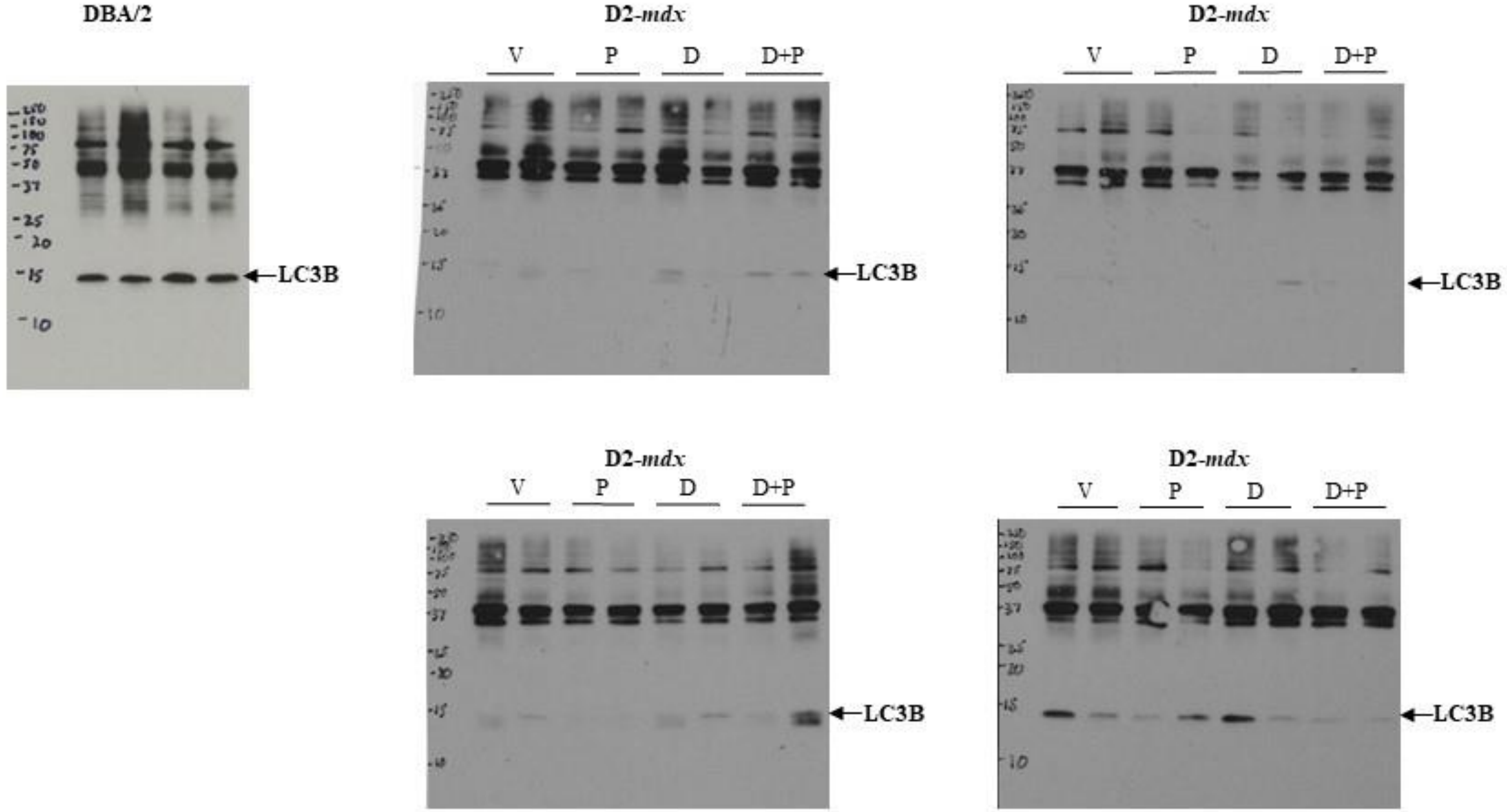

Appendix H. Western immunoblots for p62 (62kDa) in DBA/2 and treated D2-*mdx* hearts.
V=Vehicle, P=Perindopril, D=Debio-025, D+P=Debio-025+Perindopril

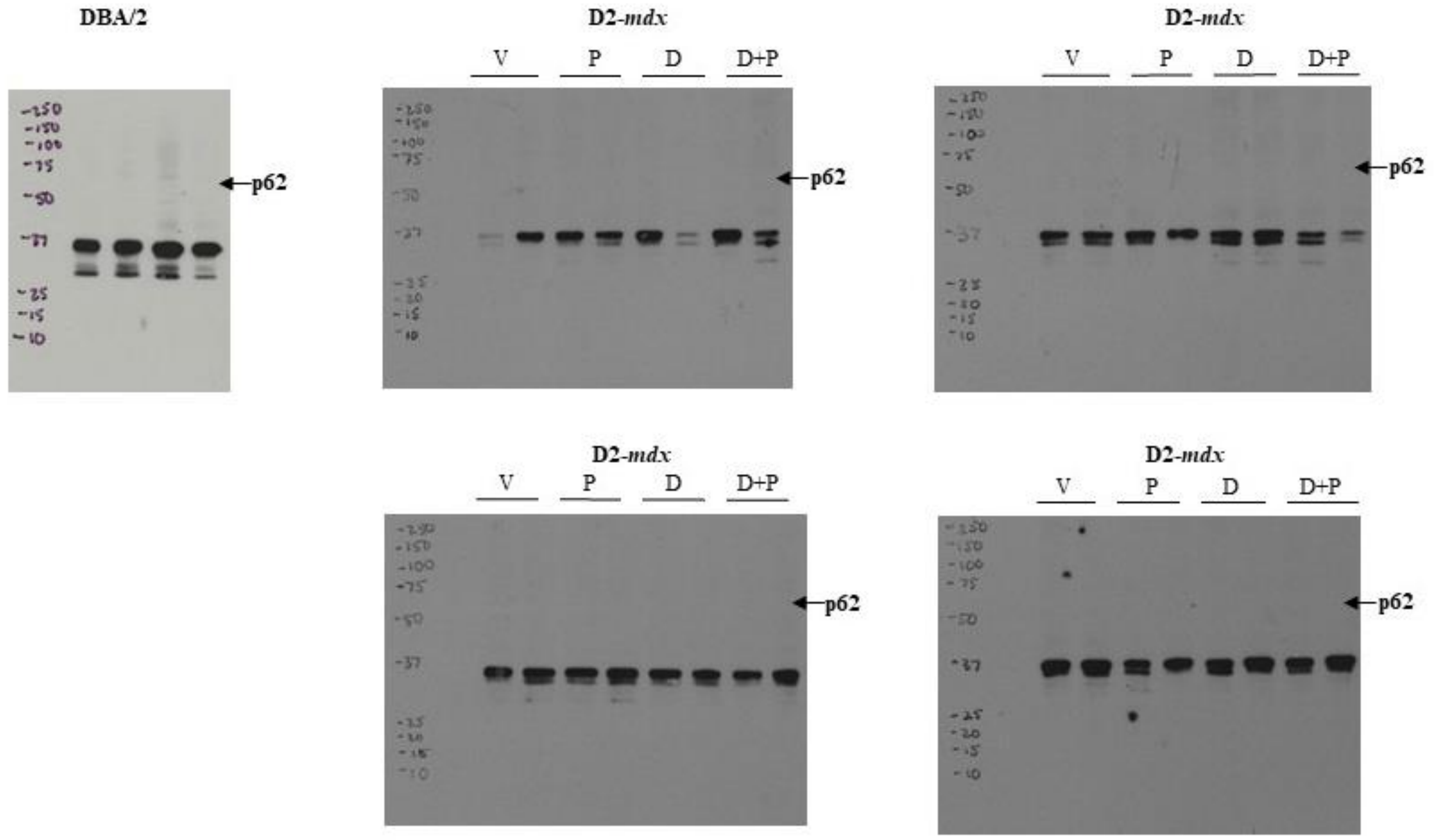

Appendix I. Western immunoblots for NF-κB (50kDa) in DBA/2 and treated D2-*mdx* hearts.
V=Vehicle, P=Perindopril, D=Debio-025, D+P=Debio-025+Perindopril

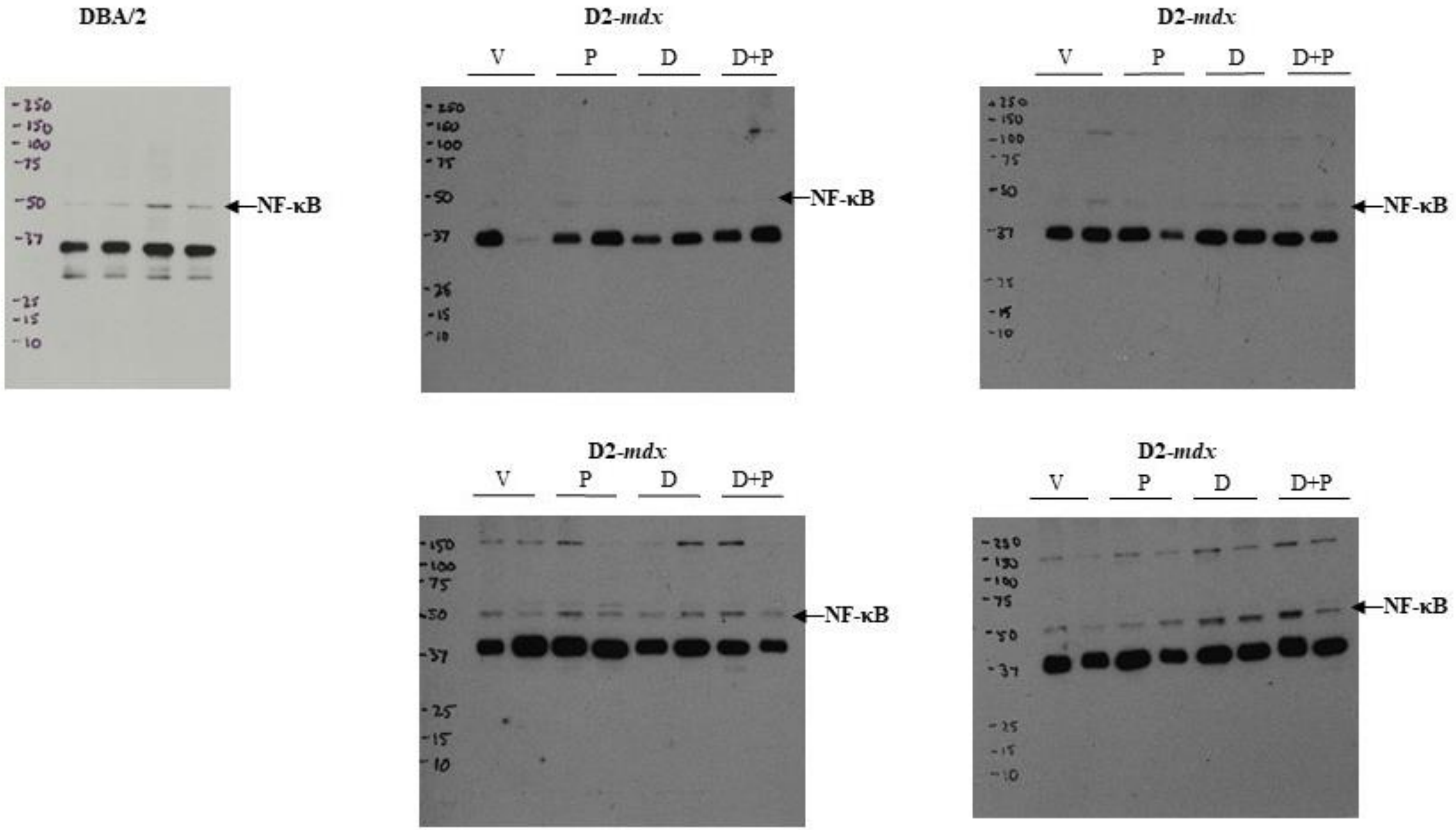

Appendix J. Western immunoblots for TNF-α (25kDa) in DBA/2 and treated D2-*mdx* hearts.
V=Vehicle, P=Perindopril, D=Debio-025, D+P=Debio-025+Perindopril

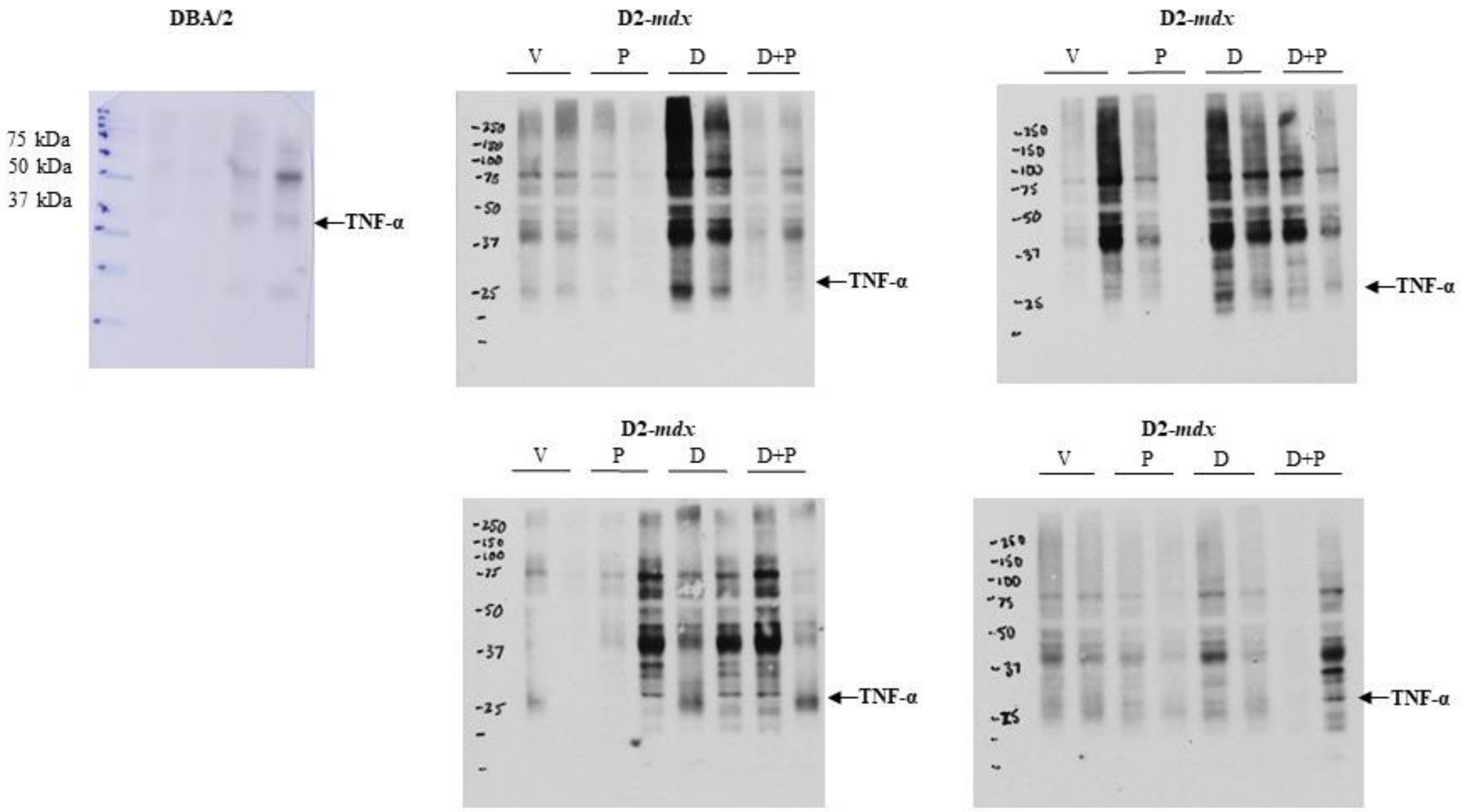

Appendix K. Western immunoblots for monoamine oxidase A (60kDa) in DBA/2 and treated D2-*mdx* hearts.
V=Vehicle, P=Perindopril, D=Debio-025, D+P=Debio-025+Perindopril

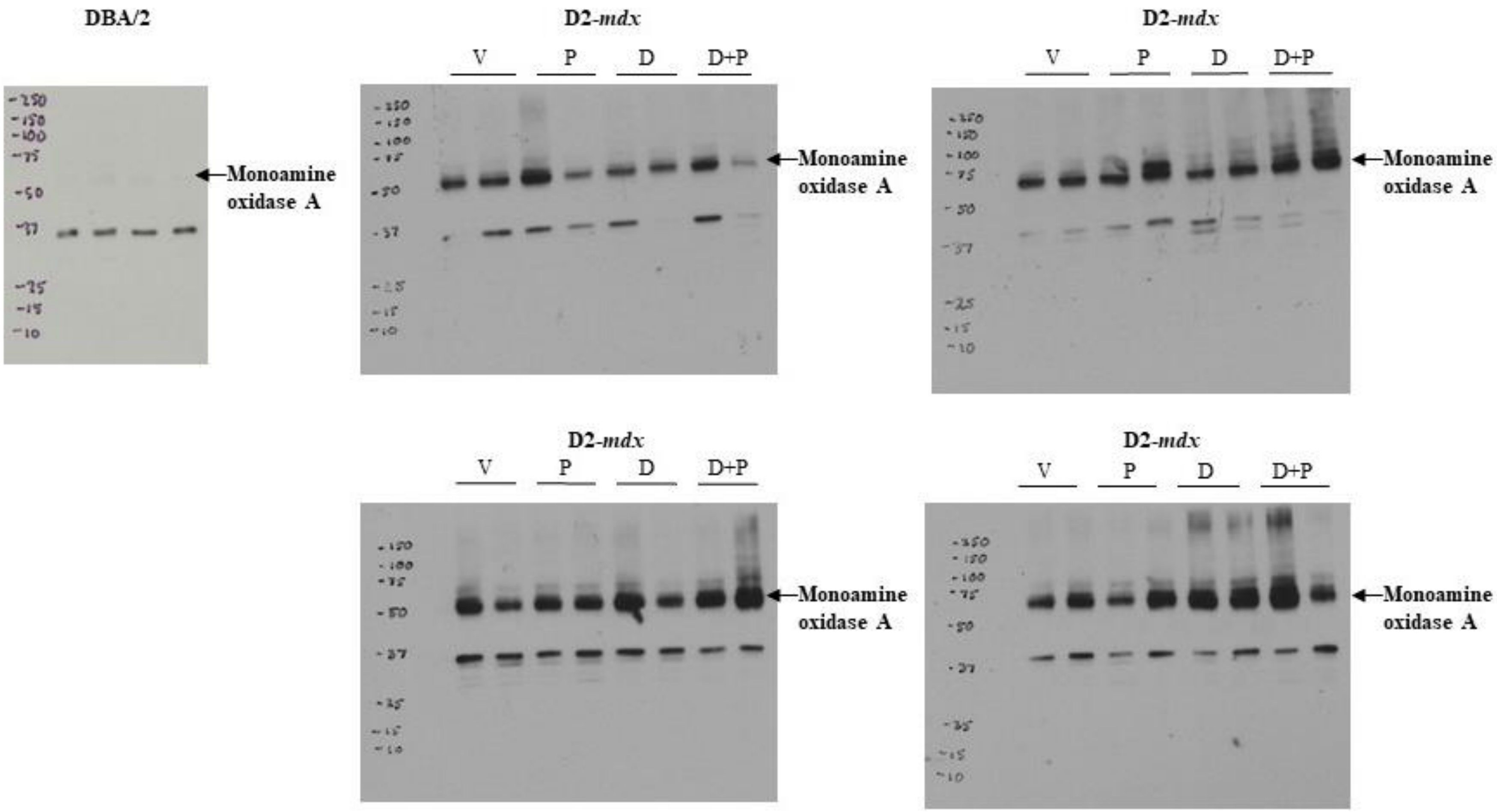

Appendix L. Western immunoblots for monoamine oxidase B (60kDa) in DBA/2 and treated D2-*mdx* hearts.
V=Vehicle, P=Perindopril, D=Debio-025, D+P=Debio-025+Perindopril

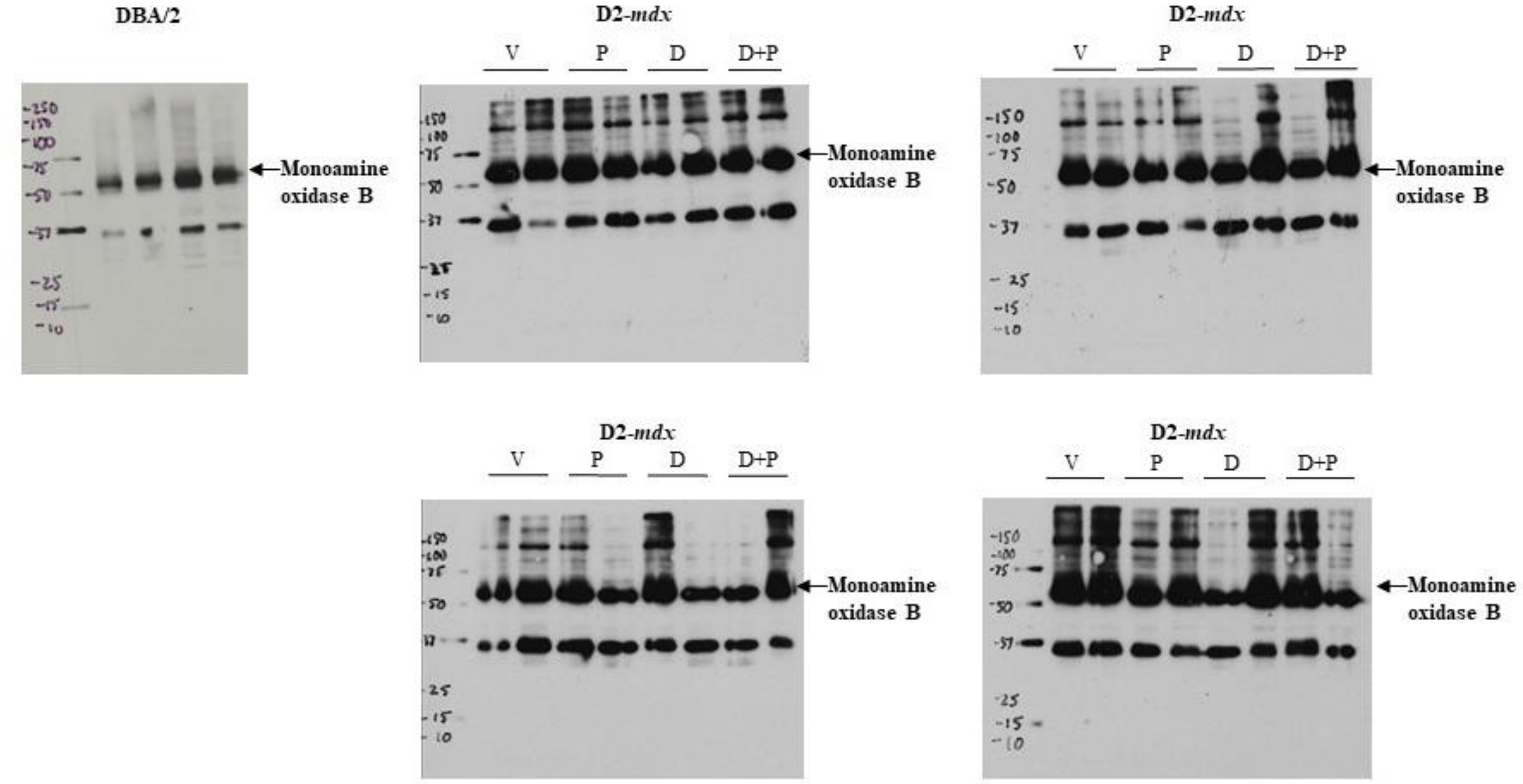

Appendix M. Examples of coomassie blue staining gels.
V=Vehicle, P=Perindopril, D=Debio-025, D+P=Debio-025+Perindopril

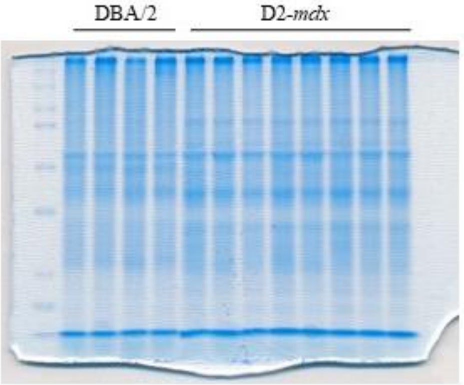

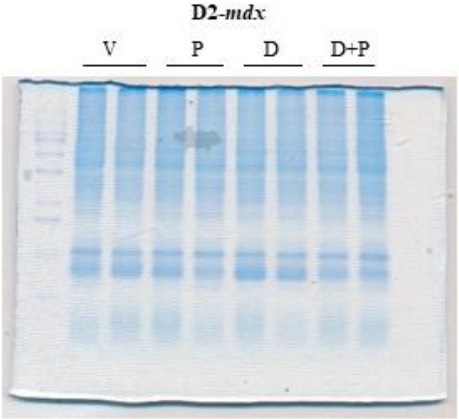

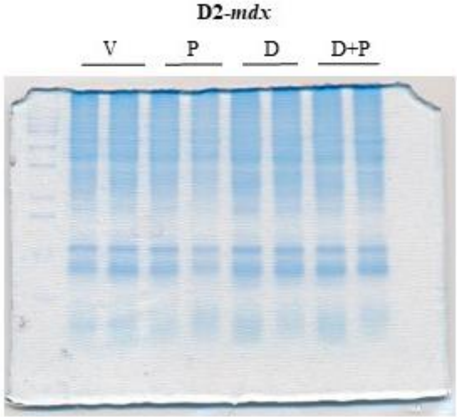

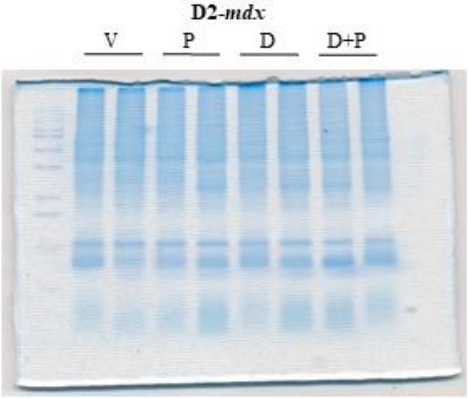

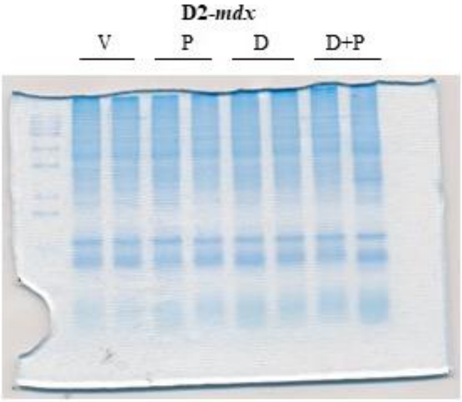

Supplement: Supplementary Materials — Western blot protein expression assays (Supplement 1). [file 5362115.f1.pdf]
